# The *Medicago truncatula* nodule identity gene *MtNOOT1* is required for coordinated apical-basal development of the root

**DOI:** 10.1186/s12870-019-2194-z

**Published:** 2019-12-19

**Authors:** Defeng Shen, Olga Kulikova, Kerstin Guhl, Henk Franssen, Wouter Kohlen, Ton Bisseling, René Geurts

**Affiliations:** Department of Plant Science, Laboratory of Molecular Biology, Wageningen University, Droevendaalsesteeg 1, 6708 PB Wageningen, The Netherlands

**Keywords:** *Medicago truncatula*, NOOT1, NOOT-BOP-COCHLEATA-LIKE, NBCL, Xylem cell differentiation, Rhizobium susceptible zone, NIN

## Abstract

**Background:**

Legumes can utilize atmospheric nitrogen by hosting nitrogen-fixing bacteria in special lateral root organs, called nodules. Legume nodules have a unique ontology, despite similarities in the gene networks controlling nodule and lateral root development. It has been shown that *Medicago truncatula NODULE ROOT1* (*MtNOOT1*) is required for the maintenance of nodule identity, preventing the conversion to lateral root development. *MtNOOT1* and its orthologs in other plant species -collectively called the NOOT-BOP-COCH-LIKE (NBCL) family- specify boundary formation in various aerial organs. However, *MtNOOT1* is not only expressed in nodules and aerial organs, but also in developing roots, where its function remains elusive.

**Results:**

We show that *Mtnoot1* mutant seedlings display accelerated root elongation due to an enlarged root apical meristem. Also, *Mtnoot1* mutant roots are thinner than wild-type and are delayed in xylem cell differentiation. We provide molecular evidence that the affected spatial development of *Mtnoot1* mutant roots correlates with delayed induction of genes involved in xylem cell differentiation. This coincides with a basipetal shift of the root zone that is susceptible to rhizobium-secreted symbiotic signal molecules.

**Conclusions:**

Our data show that *MtNOOT1* regulates the size of the root apical meristem and vascular differentiation. Our data demonstrate that *MtNOOT1* not only functions as a homeotic gene in nodule development but also coordinates the spatial development of the root.

## Background

Legume plants (Fabaceae) can form unique lateral root organs to host nitrogen-fixing rhizobium bacteria, known as nodules. Legume nodules originate from root cells upon rhizobium-induced lipo-chitooligosaccharide (LCO) signalling. In the legume model *Medicago truncatula* (medicago) LCO signalling induces cell divisions in the pericycle and endodermis, followed by a coordinated mitotic activation of cortical cells. This will give rise to nodule primordia [1]. When fully developed, a medicago nodule possesses a large central zone with cells harbouring nitrogen-fixing rhizobia, surrounded by peripheral vascular bundles and a meristem at the apex allowing indeterminate growth [1].

Legume nodule formation is controlled by a network of transcriptional regulators, among which NODULE INCEPTION (NIN) is a master regulator [2, 3]. *NIN* expression is activated upon LCO signalling in a small zone of the root with elongating root hairs [4–7]. Expression of *NIN* in the root pericycle and dividing cortical cells is essential and sufficient to trigger nodule organogenesis [6, 8, 9]. Legumes recruited a BTB/POZ-ankyrin domain containing protein of the NOOT-BOP-CHOCLEATA-LIKE (NBCL) family to maintain nodule identity in the newly formed primordium [10–12]. Knockout mutations in this gene – in medicago named *NODULE ROOT1* (*MtNOOT1*)- cause a homeotic switch from nodule organogenesis towards lateral root formation [10]. This underlines the important functioning of MtNOOT1 in nodule development. Besides nodules, *MtNOOT1* is also expressed in young root tissue [13, 14]. However, its functioning during root development remains elusive.

*MtNOOT1* is orthologous to the *Arabidopsis thaliana* (arabidopsis) *BLADE-ON-PETIOLE1* (*AtBOP1*) and *AtBOP2* genes. Studies in arabidopsis have revealed that BOP proteins function as co-transcriptional regulators involved in plant boundary formation (Reviewed in [15–19]). For example, AtBOP1 and AtBOP2 can promote expression of *LATERAL ORGAN BOUNDARIES* (*LOB*) genes to repress brassinosteroid signalling, which subsequently restricts cell growth and division in the boundary domain between the shoot apical meristem and lateral organs such as leaves [20, 21]. *AtBOP1* and *AtBOP2* also control proximal-distal leaf patterning by repressing the expression of genes that promote meristematic activity [22, 23]. Knockout mutations in *AtBOP1/AtBOP2* lead to ectopic outgrowths of blade tissue along the petioles of cotyledons and leaves, due to misexpression of meristematic genes [22, 23]. Additionally, AtBOP1 and AtBOP2 are essential for abscission zone (AZ) formation at the junction between the leaving organ and the main plant body [24]. In line with this, a complete loss of floral organ abscission is observed in the arabidopsis *Atbop1;Atbop2* double mutant [24].

Similar to arabidopsis, mutations in orthologous *BOP* genes in the legumes medicago *MtNOOT1*, pea (*Pisum sativum*) *COCHLEATA1* (*PsCOCH1*), and lotus (*Lotus japonicus*) *LjNBCL1* affect leaf patterning and AZ formation [25]. Arabidopsis *Atbop1;Atbop2* double mutants do not form stipules [24, 26], which are also simplified or reduced in the medicago *Mtnoot1* mutant and at early nodes of the pea *Pscoch1* mutant [10]. In the lotus *Ljnbcl1* mutant, nectary glands (proposed modified stipules) are completely absent [11]. Furthermore, in the medicago *Mtnoot1*, pea *Pscoch1* and lotus *Ljnbcl1* mutants, the abscission of petals is impaired [25], similar to what is observed in arabidopsis *Atbop1;Atbop2* [24]. This indicates that the function of NBCL proteins in boundary specification in the proximal region of the leaf and in AZ formation is well-conserved. In addition, MtNOOT1 and PsCOCH1 function during the root nodule development by defining the boundary between nodule meristem and nodule vasculature [10]. Taken together, NBCL proteins play a conserved role in defining boundaries in various developmental contexts.

Studies on *NBCL* genes in roots are limited. In arabidopsis it was shown that AtBOP1 and AtBOP2 play a negative role in differentiation of lignified fibres in hypocotyl and tap root [27, 28]. In arabidopsis and medicago, *AtBOP1*, *AtBOP2* and *MtNOOT1* are expressed also in the root tip [13, 14, 28], though their functioning in root development has not been unveiled yet. Here, we examined the function of *MtNOOT1* in primary root development. We show that *MtNOOT1* is involved in defining the position of the transition zone between the apical meristem and the elongation/differentiation zone, and is required for coordinated development of the root along the apical-basal axis.

## Results

### The primary root of the medicago *Mtnoot1* mutant is longer

**Fig. 1:**
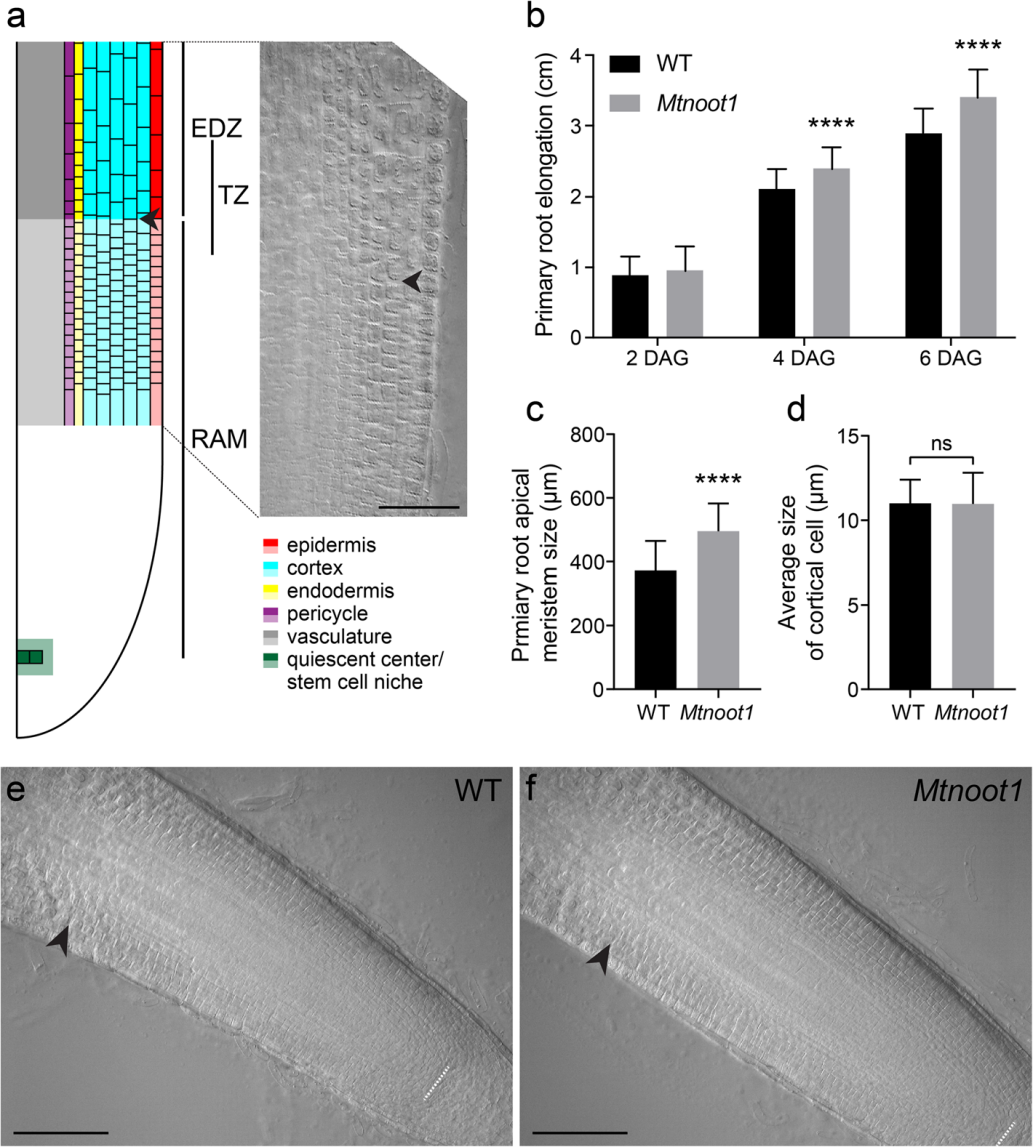
The primary root of the medicago *Mtnoot1* mutant is longer than wild-type root. **a** Schematic representation of the zonation in a medicago primary root. Left panel: the size of the root apical meristem (RAM) is determined from the stem cell niche till the elongation/differentiation zone (EDZ). The apical border of the EDZ is defined by the first elongated cortex cell of second cortical layer, as indicated by arrowhead. Note that the exact position of the RAM-EDZ boundary can vary depending on the cell type, marking a region that named transition zone (TZ) [30, 31]. Cell types are indicated by colour coding. Right panel: Normaski image of the distal region of the RAM and apical region of the EDZ of a primary root at 6 Days After Germination (DAG). Arrowhead indicates the boundary between the RAM and the EDZ of the second layer of cortical cells. **b** Root length of the medicago *Mtnoot1* tnk507 mutant is markedly longer at 4 DAG and 6 DAG when compared to primary roots of wild-type medicago seedlings (WT). **c** The RAM of medicago *Mtnoot1* tnk507 seedlings is larger at 6 DAG when compared to the RAM of primary roots of wild-type medicago seedlings. The data represent means + SD of three independent experiments, each experiment contains 16–20 roots. **d** The cortical cell size at the proximal part of the RAM is not affected in the root of the medicago *Mtnoot1* tnk507 mutant seedlings. Given is the average cell size of cortical cells at the proximal part of the RAM of wild-type and *Mtnoot1* mutant roots. The data represent means + SD of two independent experiments, each experiment contains 16–20 roots. Student *t*-test was performed to assess significant differences (****: *P* < 0.0001, ns: not significant). A representation of the RAM of wild-type (**e**) and *Mtnoot1* mutant seedling (**f**) primary roots at 6 DAG. White dotted line marks the quiescent center/stem cell niche region, arrowhead marks the boundary between the RAM and the EDZ of the second layer of cortical cells. Scale bar: 50 μm (**a**), 100 μm (**e**, **f**)

According to *Medicago truncatula* Gene Expression Atlas [29], the *MtNOOT1* gene (Medtr7g090020) is highest expressed in root tips, surpassing the expression in many nodule samples that have been analysed (Additional file 1: Figure S1). This suggests a non-symbiotic function of *MtNOOT1* in the root. To investigate this, we compared wild-type and *Mtnoot1* mutant seedlings (tnk507) when grown in vitro, and observed that the growth of the *Mtnoot1* primary root is accelerated compared to wild-type (Additional file 2: Figure S2). To obtain insight in the timing of the increase in primary root growth of the *Mtnoot1* mutant, we measured the root length at different time points (2 Days After Germination (DAG), 4 DAG and 6 DAG). This showed that the primary roots of the *Mtnoot1* mutants are markedly longer at 4 and 6 DAG when compared to wild-type seedlings (Fig. 1a-b). At 2 DAG no differences in root length was detected, suggesting that the observed differences in root length of the *Mtnoot1* mutant is not due to an earlier or faster germination. A similar result was obtained by analysing a second *Mtnoot1* mutant allele (NF2717) [10, 12] (Additional file 3: Figure S3a).

Studies in arabidopsis show that accelerated primary root growth can correlate with increased length of the root apical meristem (RAM) [30, 31]. Therefore, we compared the length of the RAM of medicago wild type and *Mtnoot1* seedlings. A difference in primary root elongation is visible at 4 DAG, which was more significant at 6 DAG (Fig. 1b). As 6 DAG is the latest time point when lateral roots have not yet emerged, we focused on this timepoint to analyse the length of the RAM. Since both *Mtnoot1* mutant lines showed a similar root length phenotype, our analysis was focused on a single mutant allele; tnk507. We found that the RAM of this *Mtnoot1* mutant is significantly larger than that of wild-type seedlings (Fig. 1c). A larger RAM can be the result of an increase in cell number or an increase in cell length. To quantify cell numbers in the medicago RAM is technically difficult due to the relatively thick root when compared to arabidopsis. To distinguish between both scenarios that can cause larger RAM, we therefore measured the length of 10 cortical cells at the proximal part of the RAM. This is the upper limit of cell number that we can confidently measure. We found that the average size of cortical cells in the RAM is not affected by the *Mtnoot1* mutation (Fig. 1d). This suggests that an increase in cell number in the RAM of the *Mtnoot1* mutant underlies its accelerated growth. Taken together, these results suggest that MtNOOT1 either represses cell division in the RAM or/and promotes cell differentiation in the transition zone of the root.

### *MtNOOT1* is expressed in the transition zone and root vasculature

**Fig. 2:**
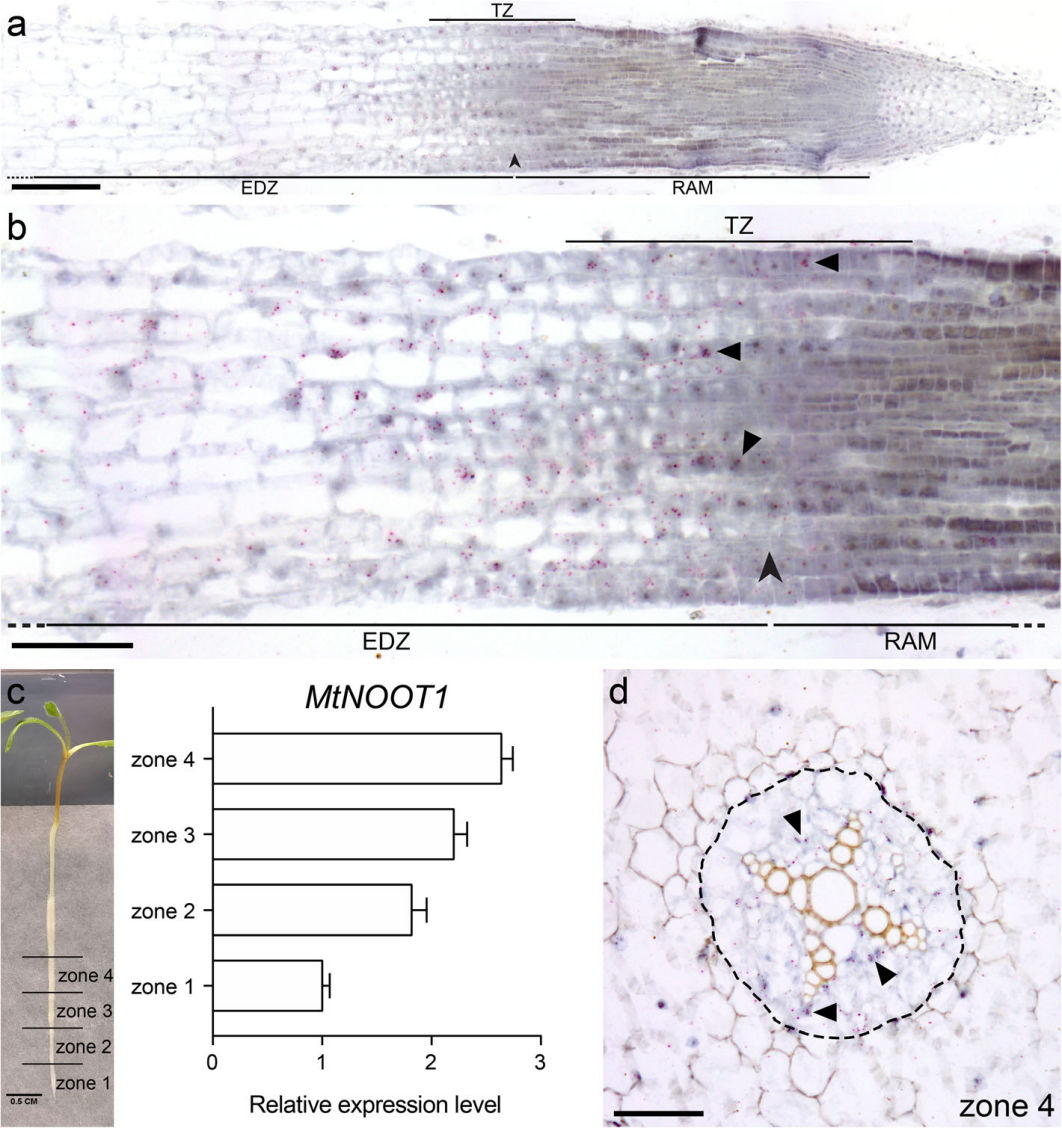
*MtNOOT1* spatial expression pattern in medicago wild-type primary root. **a, b** Longitudinal section of root tip. *MtNOOT1* expression pattern in root tip determined by in situ hybridization. *MtNOOT1* is mainly expressed in the TZ and distal part of the EDZ. **b** A close-up of (**a**), visualizing abundance of *MtNOOT1* mRNA (red dots) in epidermal and cortical cells in the TZ. Arrowhead indicates the boundary between the RAM and the EDZ of the second layer of cortical cells. **c** The expression pattern of *MtNOOT1* in different zones of root determined by qRT-PCR. Left panel: the primary root of a wild-type medicago seedling at 6 DAG is divided into four zones of 5 mm each. Right panel: *MtNOOT1* expression as determined by qRT-PCR reveals increased expression in older regions of the root. The data represent means + SEM of three independent experiments. **d** Cross section of zone 4. *MtNOOT1* expression pattern in zone 4 determined by in situ hybridization*. MtNOOT1* is mainly expressed in the procambium cells of the root vascular bundle. Black dotted line circles the pericycle of vascular bundle. Triangles indicate the *MtNOOT1* mRNA signals. Scale bar: 100 μm (**a**), 50 μm (**b**, **d**)

The accelerated primary root growth prompted us to determine the precise location of *MtNOOT1* expression. Since we were unable to identify a functional promoter region of *MtNOOT1*, we decided to perform RNA in situ hybridization on longitudinal sections of root tips (1–2 mm) of wild-type seedlings at 6 DAG. This showed that *MtNOOT1* is highly expressed in the region between the RAM and the elongation/differentiation zone and barely in the rest of the RAM (Fig. 2a). A magnification of the region with the most intense signals showed that *MtNOOT1* is expressed in the transition zone and the distal part of the elongation/differentiation zone (Fig. 2b). This suggests that MtNOOT1 controls the size of the RAM by promoting cell differentiation in the transition zone.

Since it has been shown that AtBOP1 and AtBOP2 play a role in vasculature differentiation [27, 28, 32], we questioned whether *MtNOOT1* may play a similar role in medicago roots. To investigate this, we first examined whether *MtNOOT1* is expressed in the root vasculature. We divided the first 2 cm of young seedling roots (without visible lateral roots or primordia) into four zones of each ~ 5 mm long (basipetally, from zone 1 to zone 4, of which zone 1 includes the RAM till the mature root cells with elongated root hairs). These root segments correspond to different stages of vascular development and were used to conduct qRT-PCR studies (Fig. 2c). This showed that *MtNOOT1* is expressed in all four zones, with relatively the lowest level of expression in zone 1 and the highest level in zone 4 (Fig. 2c). This is consistent with Gene Expression Atlas data (Additional file 1: Figure S1). RNA in situ hybridization on a cross section of wild-type zone 4 segment showed that *MtNOOT1* transcripts mainly occur in the procambium cells of the vasculature (Fig. 2d). This suggests that MtNOOT1 could also play a role in root vasculature development in medicago.

### The primary root of the *Mtnoot1* mutant is delayed in xylem cell differentiation

**Fig. 3:**
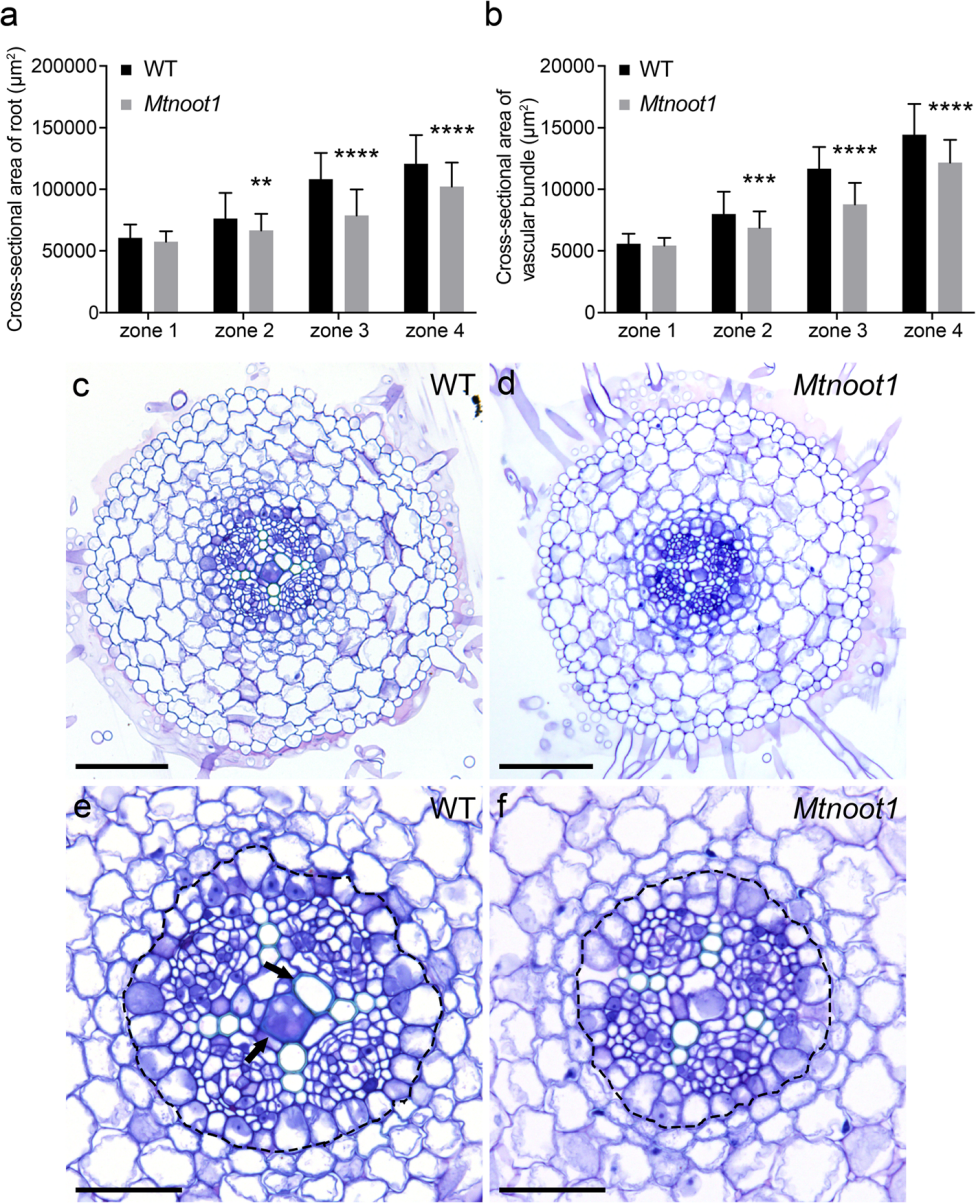
Medicago *Mtnoot1* mutant seedlings have a thinner primary root at 6 DAG. The size of the cross-sectional area of (**a**) primary root and (**b**) vascular bundle of the medicago *Mtnoot1* tnk507 mutant at zone 2, 3 and 4 are significantly smaller when compared to wild-type medicago roots. The data represent means + SD of three independent experiments, each experiment contains 18–20 roots. Student *t*-test was performed to assess significant differences (**: *P* < 0.01, ***: *P* < 0.001, ****: *P* < 0.0001). Representative root cross sections of wild-type (**c**) and *Mtnoot1* (**d**) roots, and wild-type (**e**) and *Mtnoot1* (**f**) vascular bundle at zone 4. Black dotted line circles the pericycle of vascular bundle. Black arrow marks differentiated/lignified metaxylem cells, which are not found in the *Mtnoot1* mutant. Scale bar: 100 μm (**c**, **d**), 50 μm (**e**, **f**)

As *MtNOOT1* is expressed in the root vasculature, we investigated whether the *Mtnoot1* mutant is affected in root vasculature development. Preliminary observations suggested that *Mtnoot1* mutant roots are thinner than wild-type roots. To quantify this, cross sections on *Mtnoot1* mutant line tnk507 were made on the middle parts of zone 1 to zone 4 and the size of the cross-sectional area was determined. This showed that in zone 2 to zone 4 the cross-sectional area of the *Mtnoot1* mutant roots is significantly smaller when compared to the counterparts of wild-type roots. The root cross-sectional area sizes of zone 3 and zone 4 of the *Mtnoot1* mutants were more comparable to those of zone 2 and zone 3 in wild-type, respectively (Fig. 3a). A similar reduction in root cross-sectional area size was also observed in the *Mtnoot1* mutant line NF2717 (Additional file 3: Figure S3b). Further, the cross-sectional area sizes of the vasculature also showed that this is significantly smaller in *Mtnoot1* (tnk507) root zone 2 to zone 4 when compared to wild-type roots. This suggests that the *Mtnoot1* mutants have a delayed pattern of root radial growth, which correlates with the growth defects in its vasculature.

**Fig. 4:**
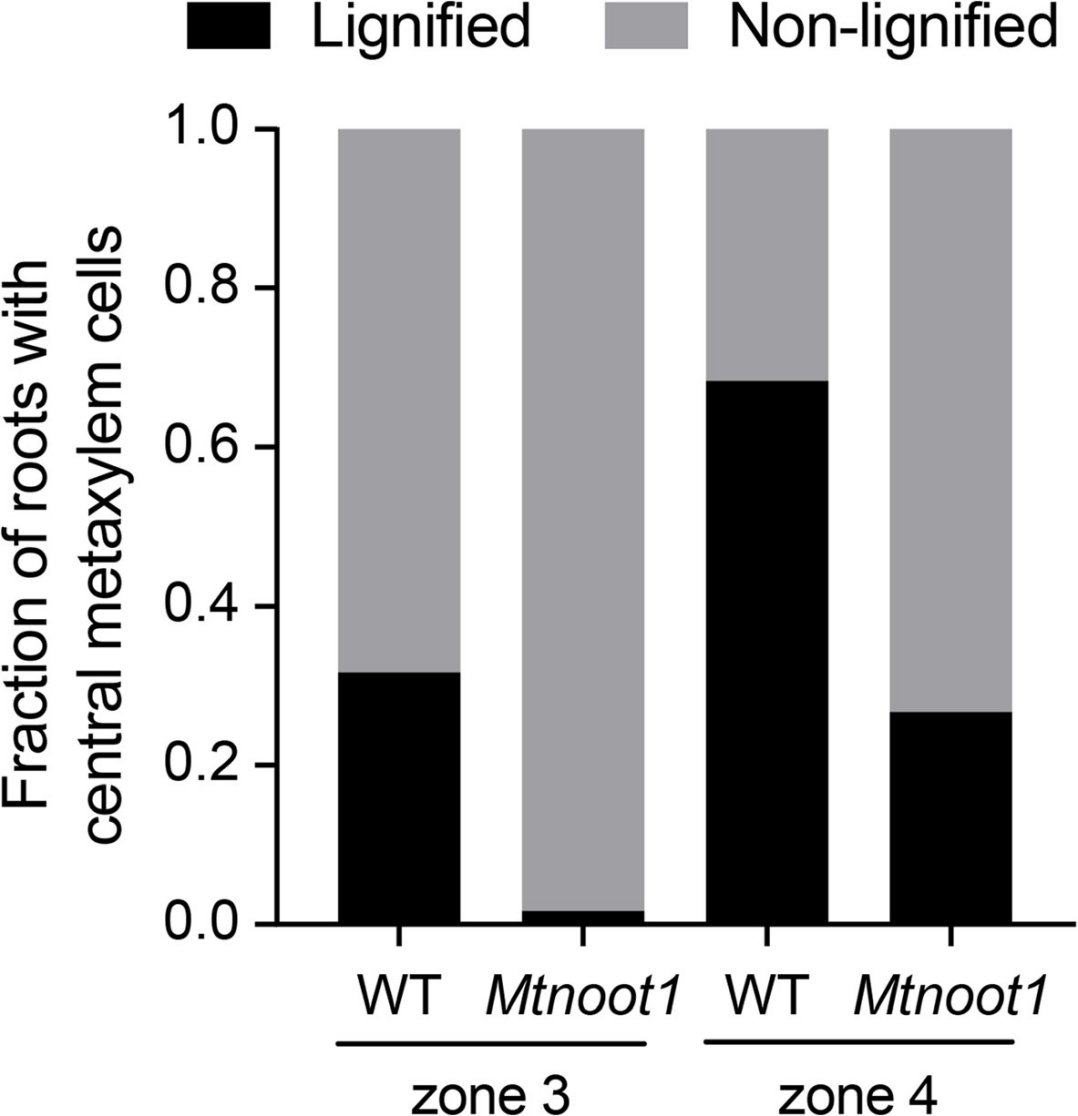
Vascular xylem differentiation is delayed in the primary root of *Mtnoot1* at 6 DAG. The fraction of roots with lignified central metaxylem cells in zone 3 and zone 4 is decreased in the *Mtnoot1* tnk507 mutant when compared with wild-type seedlings. The lignification level of the central metaxylem cells in zone 4 of the *Mtnoot1* mutant is similar to that of wild-type medicago zone 3, indicating that the differentiation of vascular xylem cells is delayed in the *Mtnoot1* mutant. The presented data combines three independent experiments, each experiment contains 20 roots

Vascular bundles are mainly built up of xylem and phloem cells, in medicago roots in a four-arch constitution (Fig. 3c, e). During the development of the root, vascular metaxylem cells differentiate and become lignified [33]. Upon toluidine blue staining, lignified metaxylem cells gain a lighter blue colouration than non-lignified cells [34]. Upon toluidine blue staining of wild-type and *Mtnoot1* tnk507 roots, we found that 32% (19/60) of wild-type zone 3 showed lignified central metaxylem cells, in contrast to only 2% (1/60) of *Mtnoot1* zone 3 showed lignification of central metaxylem cells. Furthermore, central metaxylem cells are lignified in 68% (41/60) of wild-type zone 4, but only in 27% (16/60) of *Mtnoot1* zone 4, which is more similar to the lignification level of wild-type zone 3 (Fig. 4). A similar result was obtained by analysing the *Mtnoot1* NF2717 mutant allele (Additional file 3: Figure S3c-g). These observations demonstrate that in *Mtnoot1* mutants the lignification level of vascular metaxylem cells in zone 3 and zone 4 is lower when compared with wild-type, suggesting that the differentiation of *Mtnoot1* root vasculature is delayed. This is consistent with the observation that the cross-sectional area sizes of *Mtnoot1* root vasculature in zone 2 to zone 4 are significantly smaller when compared to wild-type (Fig. 3b).

### The expression of genes involved in xylem cell differentiation is delayed in *Mtnoot1* mutant roots

**Fig. 5:**
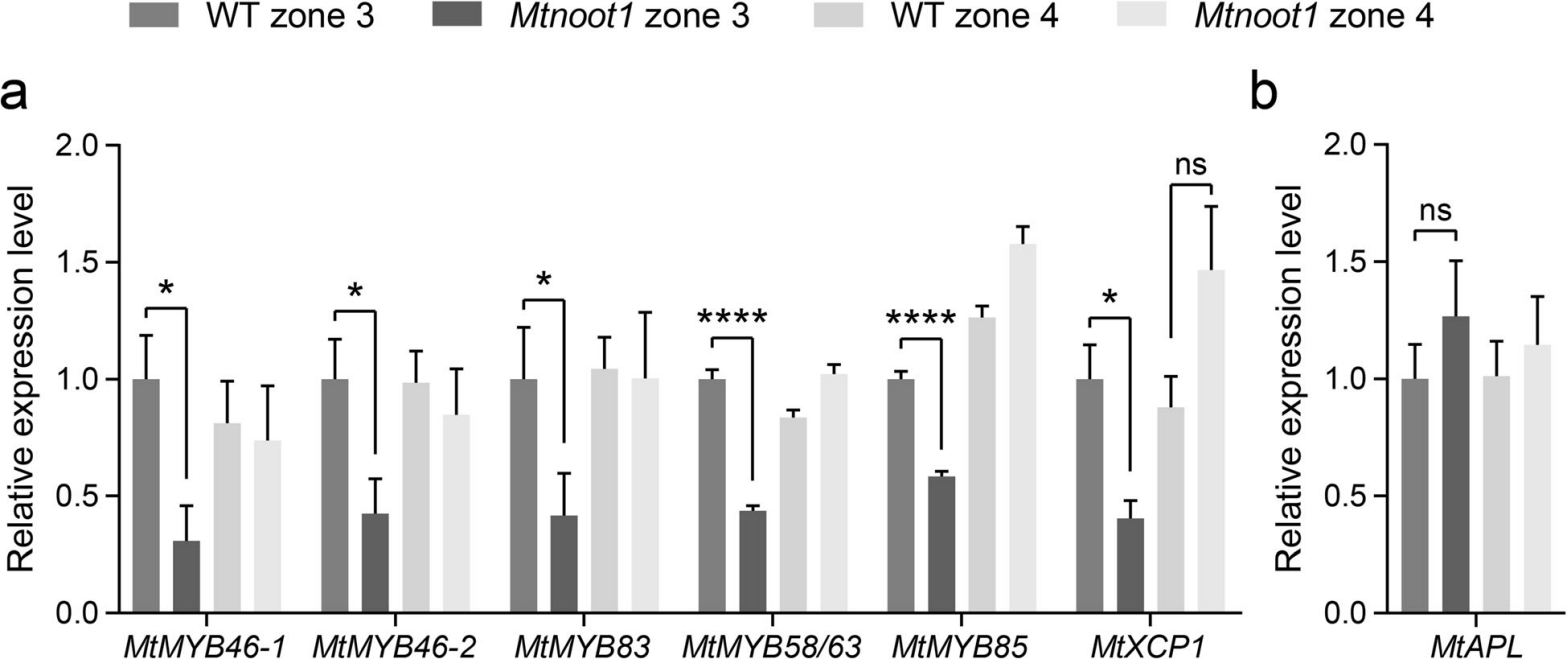
Genes putatively involved in xylem cell differentiation display reduced expression in *Mtnoot1* roots. **a, b** Expression level of various genes putatively involved in xylem and phloem cell differentiation in zone 3 and zone 4 of wild-type and *Mtnoot1* tnk507 roots determined by qRT-PCR. **a** The MYB-type transcription factors *MtMYB46–1*, *MtMYB46–2*, *MtMYB83* and *MtMYB85*, and the XYLYME CYSTEINE PEPTIDASE 1 putative ortholog *MtXCP1* are lower expressed in zone 3 of *Mtnoot1* mutant roots when compared to wild-type. **c** The expression level of phloem marker gene *MtAPL* is not affected in *Mtnoot1* mutant roots. The data represent means + SEM of two (*MtXCP1* and *MtAPL*) or three (*MtMYBs*) independent experiments. Student *t*-test was performed to assess significant differences (*: *P* < 0.05, ****: *P* < 0.0001, ns: not significant)

In arabidopsis, secondary cell wall formation and subsequent programmed cell death (PCD) are two critical steps in the maturation of proto−/metaxylem and fibre cells [33]. Secondary cell wall biosynthesis is initiated by the master regulators VASCULAR-RELATED NAC-DOMAIN 7 (AtVND7) for protoxylem, AtVND6 for metaxylem and SECONDARY WALL–ASSOCIATED NAC DOMAIN PROTEIN 1 (AtSND1) for fibre differentiation [33]. These master regulators control secondary cell wall biosynthesis via a network of genes, which include the transcription factors *AtMYB46, AtMYB58, AtMYB63, AtMYB83* and *AtMYB85* that ultimately control lignin biosynthesis genes, and the peptidase encoding gene *XYLEM CYSTEINE PEPTIDASE 1* (*AtXCP1*) involved in the PCD during xylem cell development [33, 35–37]. To find molecular support for the observation of the delayed vasculature development in the *Mtnoot1* mutant, we aimed to analyse the transcript levels of the putative medicago orthologues of the above-mentioned genes. To identify such medicago orthologues we used phylogenetic reconstruction (Additional files 4-9: Figures. S4-S9). Subsequent qRT-PCR expression studies on medicago root zone 3 and zone 4 revealed that both *MtVND6* and *MtVND7*, but not *MtSND1* are slightly lower expressed in zone 3 of *Mtnoot1* mutant root (Additional file 10: Figure S10), while the putative downstream targets *MtMYB46–1*, *MtMYB46–2*, *MtMYB83*, *MtMYB58/63*, and *MtMYB85* and *MtXCP1* are markedly lower expressed in the *Mtnoot1* root (Fig. 5a). In zone 4 of the *Mtnoot1* mutant root, the expression of all these genes was restored to the wild-type level (Fig. 5a), indicating a delayed transcriptional regulation of genes controlling secondary cell wall biosynthesis in the apical-basal direction in the *Mtnoot1* mutant roots. In contrast, the expression level of a phloem marker gene, *ALTERED PHLOEM DEVELOPMENT* (*MtAPL*) [38], is not affected in *Mtnoot1* (Fig. 5b), suggesting that phloem cell development is not disturbed. Taken together, these results support our observation that xylem cell differentiation in the *Mtnoot1* mutant is delayed.

### The LCO susceptible zone of *Mtnoot1* mutant roots is shifted basipetally

**Fig. 6:**
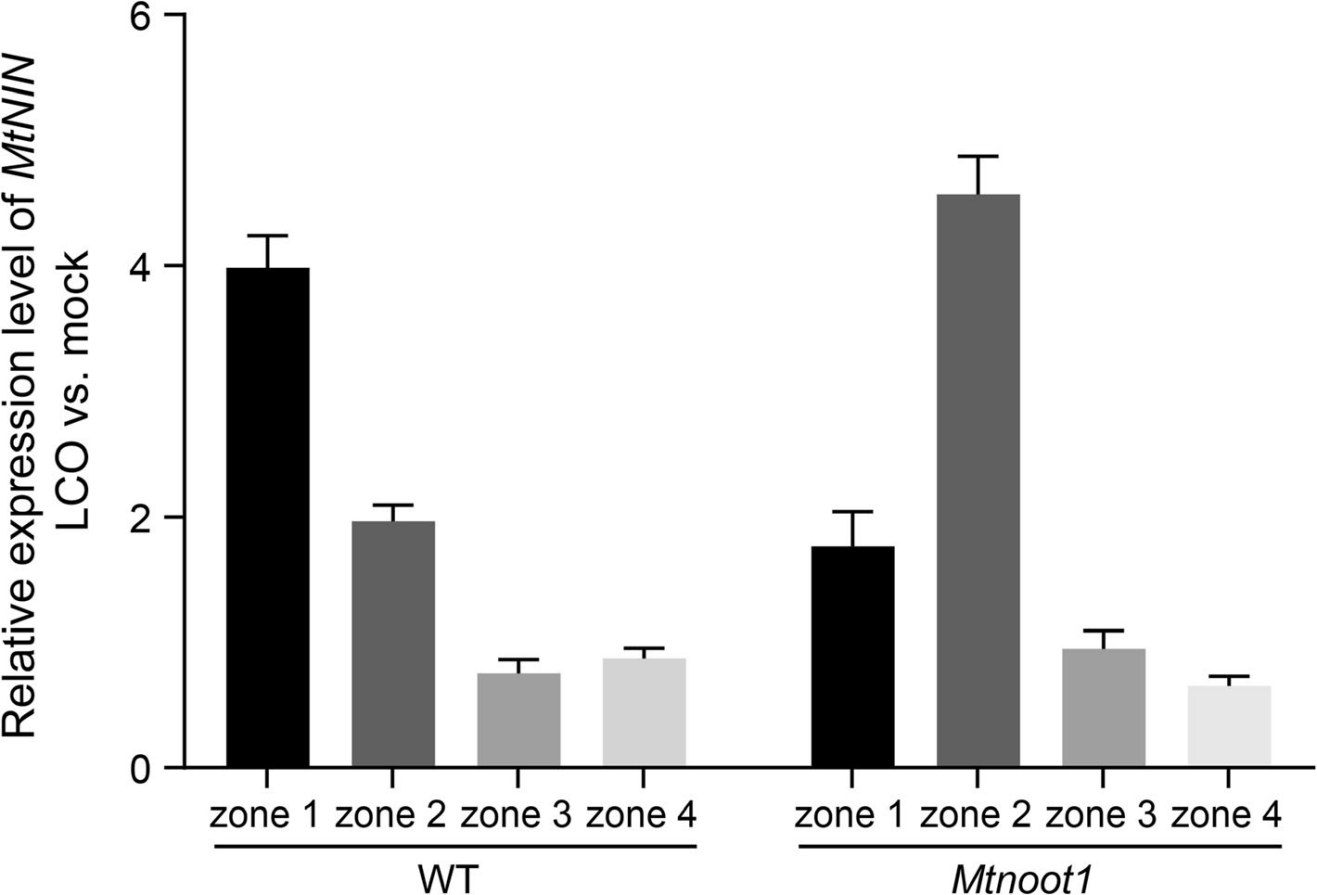
Rhizobium LCO-induced *MtNIN* expression is spatially different in the medicago *Mtnoot1* roots in comparison with wild-type roots. *MtNIN* expression is quantified using qRT-PCR in four root zones at three hours post application of 10^− 9^ M *S. meliloti* LCOs. The data represent means + SEM of two independent experiments using the *Mtnoot1* tnk507 mutant

In medicago, the site where the interaction with rhizobia occurs is tightly linked to the developmental status of the root tissue. At the start of the differentiation zone, where young elongating root hairs can be found is called the susceptible zone, and it is here where rhizobium LCOs can trigger expression of symbiotic genes such as *MtNIN* [5–7]. The delayed root differentiation observed in the medicago *Mtnoot1* mutant led us to question whether this may also affect the susceptibility of the root to rhizobium. To investigate this, we studied the expression of *MtNIN* in the four root zones upon application of ~ 10^− 9^ M *Sinorhizobium meliloti* 2011 LCOs (dissolved in 1% DMSO) or mock (1% DMSO). qRT-PCR expression analysis showed that in wild-type roots the strongest induction of *MtNIN* occurred in zone 1 and a lower induction in zone 2. This indicates that the LCO susceptible zone of wild-type predominately locates in zone 1 (Fig. 6). In contrast, in the medicago *Mtnoot1* mutant, the strongest *MtNIN* expression is observed in zone 2 (Fig. 6), implying that the *Mtnoot1* mutation caused the LCO susceptible zone to shift to zone 2. This is consistent with the delayed differentiation of the *Mtnoot1* root, which affects the LCO response by basipetally shifting the LCO susceptible zone.

## Discussion

Here, we demonstrate that *MtNOOT1* is required for coordinated development of the primary root of medicago along the apical-basal axis. A knockout mutation in the *MtNOOT1* gene causes two root developmental affects; (1) the primary root growth is faster due to a larger RAM, meaning more dividing cells, and (2) the primary root is thinner with delayed root differentiation. This demonstrates that in roots *MtNOOT1* not only functions as a homeotic gene in nodule development, but also coordinates root development.

In situ hybridization revealed that *MtNOOT1* is expressed in the transition zone, which is located between the RAM and the elongation/differentiation zone. A larger RAM due to an increased number of meristematic cells indicates that the transition zone is positioned more distal from the stem cell niche. This suggests that MtNOOT1 controls the size of the RAM by defining the position of the boundary region (i.e. the transition zone) between the RAM and the elongation//differentiation zone. This is consistent with the aerial function of its orthologs in arabidopsis, where AtBOP1 and AtBOP2 promote the boundary specification between groups of cells with different fates [15–18]. In contrast to what we report for medicago *Mtnoot1*, a root growth and differentiation phenotype has not been reported in the arabidopsis *Atbop1;Atbop2* double mutant [28]. The recruitment of NBCL family by legumes to maintain nodule symbiotic organ identity by defining nodule territories may have required adaptations in protein regulation and functioning [10]. Such adaptations could be causal for the difference in root functioning of MtNOOT1 in medicago and AtBOP1 and AtBOP2 in arabidopsis. Further, it has been proposed that arabidopsis has a closed RAM organization with distinct initial cells, while legumes have a RAM with a basic-open organization. In the latter case, the identity of cell files seems disorganized and cannot be predicted [39]. This deviation in structure of the RAM may explain the different functioning of BOP1/NOOT1 in arabidopsis and legume roots.

We also noted a basipetal shift of the root zone that is susceptible for rhizobium LCO signal molecules in the medicago *Mtnoot1* mutant. Such shift of susceptibility to rhizobium can be explained by the delayed differentiation of root cells. In medicago, susceptibility to LCO signalling can only occur in defined window of development. By elongating the RAM, the differentiation zone gets shifted basipetally. As a result, the susceptible zone shifts basipetally as well.

In the arabidopsis root the transition zone is determined by the antagonistic interaction between auxin and cytokinin. A well-defined auxin minimum is established in the boundary region, which is mediated by an active cytokinin signalling in the transition zone [40]. Activation of auxin signalling or inhibition of cytokinin signalling can lead to accelerated primary root elongation and a larger RAM with increased number of meristematic cells, which are similar to the phenotype of *Mtnoot1* roots [30, 31]. Auxin and cytokinin are also involved in root vasculature development (Reviewed in [41, 42]). For example, mutations in cytokinin biosynthetic genes lead to abolished cambium formation and reduced thickening of the arabidopsis root [43], and auxin signalling is required for xylem differentiation and the organizer identity of vascular cambium cells in arabidopsis root [44]. In legumes, auxin and cytokinin participate in the LCO response and nodule organogenesis (Reviewed in [45–47]). For example, a mutation in cytokinin perception severally weakens the LCO response and perturbs nodule organogenesis [7, 48], whereas LCOs trigger auxin biosynthesis in the epidermis, leading to a rapid accumulation of auxin locally [49]. Recent findings suggest that in *Mtnoot1* nodules the interplay between auxin and cytokinin is imbalanced [12]. In line with that, we speculate that the auxin/cytokinin signalling pathway is affected during the primary growth of *Mtnoot1* roots, which will be targeted in future research.

We also showed that the lignification of xylem cells is delayed in *Mtnoot1* primary roots, which correlates with a delayed induction of a cascade of transcription factors involved in xylem cell differentiation. This can be explained by the extended RAM, leading to delayed cell differentiation in the basal region of primary root. However, the expression of *MtNOOT1* in the procambium cells by in situ hybridization suggests that MtNOOT1 can positively regulates vascular cell differentiation in a cell-autonomous manner. This is in line with the function of arabidopsis AtBOP1 and AtBOP2 in stem vasculature, where they can induce lignin deposition in vascular cells by promoting the expression of lignin biosynthetic genes. Ectopic expression of *AtBOP1/AtBOP2* in stem tissue leads to an expanded pattern of lignification [32]. Therefore, we hypothesize that MtNOOT1 promotes root vasculature differentiation by activating a cascade of genes involved in xylem cell differentiation.

## Conclusions

Here, we showed that *MtNOOT1* controls two aspects of root development; (1) the positioning of the transition zone, and (2) vasculature development by transcriptionally activating of a cascade of genes involved in xylem cell differentiation at a controlled distance from the root tip. Knockout of the *MtNOOT1* gene not only leads to a delayed differentiation of the root, but also shift the root region susceptible to LCOs. Taken together, this demonstrates that *MtNOOT1* not only maintains nodule identity, but also coordinates the primary root development along the apical-basal axis.

## Methods

### Plant materials and growth conditions

*Medicago truncatula* wild-type accession R108–1 plants and *Mtnoot1* mutant lines tnk507 and NF2717 were used in this study [10, 12]. R108–1 was acquired from Toan Hanh Trinh [50]. tnk507 and NF2717 were acquired from Pascal Ratet (IPS2, CNRS, Gif-sur-Yvette, France), they were identified by a forward genetics screen of Tnt1 insertion lines (Institut des Sciences du Végétal, France; Noble Foundation, Ardmore, USA). The surface-sterilization and germination of medicago seeds were performed as previously described by [51]. Note that medicago seeds germinated at room temperature for one day, before growing on Fahraeus agar medium [52] (including 0.75 mM Ca (NO_3_)_2_) in square petri dish (9 cm × 9 cm) and exposed to light for another five days. Plants were grown in an environmentally controlled growth chamber at 21 °C with a 16-h light/8-h dark.

### Microscopy and imaging

For microscopy studies, root segments were all collected at 6 DAG. For measuring the length of the RAM, ~ 5 mm root tips were cut and immersed in chloral hydrate solution at 4 °C overnight, analyzed under Axio Imager A1 microscope (Zeiss) with Nomarski optics. For measuring cross-sectional areas, root segments were fixed with 4% paraformaldehyde (w/v), 5% glutaraldehyde (v/v) in 0.05 M sodium phosphate buffer (pH 7.2) at 4 °C overnight. The fixed material was dehydrated in an ethanol series and subsequently embedded in Technovit 7100 (Heraeus Kulzer) according to the manufacturer’s protocol. Sections (5 μm) were made with a RJ2035 microtome (Leica Microsystems) stained 1.5 min in 0.05% toluidine blue O. For phloroglucinol-HCl staining, root segments were fixed as abovementioned. The fixed material was washed with 1 x PBS (sodium phosphate buffer), and directly embedded in 6% low melting agarose dissolved in 1 x PBS. Sections (50 μm) were made with a VT1000 S vibratome (Leica Microsystems), stained with 2% phloroglucinol (in 95% ethanol) for 2 min and applied with a few drops of 37% HCl. Sections were all analysed by using a DM5500B microscope equipped with a DFC425C camera (Leica Microsystems).

### In situ hybridization

Hybridization was performed twice on root segments at 6 DAG by using Invitrogen ViewRNA ISH Tissue 1-Plex assay kit (Thermo Fisher Scientific), as previously described by [9, 53]. For user manual, visit https://assets.thermofisher.com/TFS-Assets/LSG/manuals/MAN0018633_viewRNA_ISH_UG.pdf. The probe sets for *MtNOOT1* (catalogue number: VF1–16434, information is available on request) were designed and synthesized by Thermo Fisher Scientific. *MtNOOT1* probe sets cover the region 2–913 nucleotide (nt) of the coding sequence (1449 nt, Medtr7g090020.1). A typical probe set contains ~ 20 oligonucleotide pairs of probes that hybridize to specific regions across the target mRNA. Each probe covers 20 nt, only a pair of two adjacent probes, which can target 40 nt, can form a site for signal amplification. By this principle, control probes are not needed [9, 53–56]. Sections were imaged as mentioned above.

### Phylogenetic tree construction

The protein sequences of different orthogroups were obtained from [57]. For phylogenetic reconstruction, full length (predicted) protein sequences of at least two closely related orthogroups were aligned using MAFFT v7.017 [58], implemented in Geneious R6 (Biomatters, Auckland, New Zealand), using default parameter settings. After manual inspection, alignments were used for tree building by using W-IQ-TREE [59] with best-fit substitution model [60]. Branch support was assessed by using Ultrafast Bootstrap Approximation based on 1000 replicates [61].

### RNA isolation and qRT-PCR analysis

RNA was isolated from root segments at 6 DAG using the EZNA Plant RNA mini kit (Omega), following the supplier’s manual. 1 μg total RNA was used to synthesize cDNA using iScript cDNA synthesis kit (Bio-Rad). Equal amounts of cDNA were used for qPCR using SYBR Green Super-mix (Bio-Rad) in a Bio-Rad CFX connect real-time system qPCR machine. Cycling conditions were: 95 °C for 3 min, [95 °C for 15 s, 60 °C for 30 s] (40 cycles), 95 °C for 10 s, followed by melt curve analysis (from 65 °C to 95 °C, at an increment of 0.5 °C, for 5 s). The gene expression was normalized using *MtACT2* as reference gene. Three technical replicates per biological replicate. All primers used in this study are listed in Additional file 11: Table S1.

## Supplementary information

**Additional file 1: Figure S1**. Medicago *MtNOOT1* is expressed in the root tip. Expression profiles are derived from the *Medicago truncatula* Gene Expression Atlas [29]. *MtNOOT1* is targeted by the probe-sets Mtr.19586.1.S1_at, Mtr.27707.1.S1_s_at, and Mtr.39297.1.S1_s_at. Root 3 mm tip: 3 mm root tip [14]; adj tip: 1 cm root segment adjacent to 3 mm root tip [14]; Nod: nodules, all nodule samples are derived from [13]; dpi: days post inoculation. (DOCX)

**Additional file 2: Figure S2**. The primary root *Mtnoot1* tnk507 mutant is longer than wild-type. Representative seedlings at 6 DAG are presented. (DOCX)

**Additional file 3: Figure S3**. The *Mtnoot1* NF2717 mutant allele shows a similar phenotype as the *Mtnoot1* tnk507 allele. **a** Root length of the medicago *Mtnoot1* mutant (NF2717) is markedly longer at 4 DAG and 6 DAG when compared to primary roots of wild-type medicago seedlings (WT). **b** The cross-sectional area is significantly reduced in the medicago *Mtnoot1* mutant (NF2717) at zone 3 and zone 4. The data represent means + SD of two independent experiments, each experiment contains 15–20 roots. Student *t*-test was performed to assess significant differences (****: *P* < 0.0001). Representative root cross sections of wild-type (**c, e**) and *Mtnoot1* (NF2717) (**d, f**) vascular bundle at zone 3 stained with phloroglucinol-HCl to demonstrate lignin deposition at 6 DAG. Black arrow marks lignified metaxylem cells, which are not found in the *Mtnoot1* mutant (NF2717). **g** Vascular xylem differentiation is delayed in the *Mtnoot1* (NF2717) primary root at 6 DAG. The fraction of roots with lignified central metaxylem cells in zone 3 and zone 4 is decreased in the *Mtnoot1* mutant when compared with wild-type seedlings. The presented data combines two independent experiments, each experiment contains 15–18 roots. Scale bar: 50 μm (**c**, **d**), 100 μm (**e**, **f**). (DOCX)

**Additional file 4: Figure S4**. Maximum likelihood tree of VND6, VND7 and related proteins. The protein sequences of OG0006787 (red), OG0009959 (dark red), OG0004118 (purple), OG0001465 (green and blue) are obtained from van Velzen et al. (2018), except VND6, which was not included in OG0001465. Species include arabidopsis (Athaliana), *Eucalyptus grandis* (Egrandis), *Fragaria vesca* (Fvesca), *Glycine max* (Gmax), medicago (Mtruncatula), *Populus trichocarpa* (Ptrichocarpa), *Parasponia andersonii* (Pan) and *Trema orientalis* (Tor). Numbers at to the branches indicate support from 1000 ultrafast bootstrap replicates. OG0006787 including NAC1 was used as outgroup. (DOCX)

**Additional file 5: Figure S5**. Maximum likelihood tree of SND1 and related proteins. The protein sequences of OG0009898 (red) and OG0001875 (green) are obtained from van Velzen et al. (2018). Species include arabidopsis (Athaliana), *Eucalyptus grandis* (Egrandis), *Fragaria vesca* (Fvesca), *Glycine max* (Gmax), medicago (Mtruncatula), *Populus trichocarpa* (Ptrichocarpa), *Parasponia andersonii* (Pan) and *Trema orientalis* (Tor). Numbers at to the branches indicate support from 1000 ultrafast bootstrap replicates. OG0009898 containing SMB was used as outgroup. (DOCX)

**Additional file 6: Figure S6**. Maximum likelihood tree of MYB46, MYB83 and related proteins. The protein sequences of OG0000857 (red) and OG0001270 (green) are obtained from van Velzen et al. (2018). Species include arabidopsis (Athaliana), *Eucalyptus grandis* (Egrandis), *Fragaria vesca* (Fvesca), *Glycine max* (Gmax), medicago (Mtruncatula), *Populus trichocarpa* (Ptrichocarpa), *Parasponia andersonii* (Pan) and *Trema orientalis* (Tor). Numbers at the branches indicate support from 1000 ultrafast bootstrap replicates. OG0000857 containing MYB50 was used as outgroup. (DOCX)

**Additional file 7: Figure S7**. Maximum likelihood tree of MYB58, MYB63 and MYB85 proteins. The protein sequences of OG0005384 (red) and OG0002420 (green) are obtained from van Velzen et al. (2018). Species include arabidopsis (Athaliana), *Eucalyptus grandis* (Egrandis), *Fragaria vesca* (Fvesca), *Glycine max* (Gmax), medicago (Mtruncatula), *Populus trichocarpa* (Ptrichocarpa), *Parasponia andersonii* (Pan) and *Trema orientalis* (Tor). Numbers at the branches indicate support from 1000 ultrafast bootstrap replicates. OG0005384 containing MYB58 and MYB63 was used as outgroup. (DOCX)

**Additional file 8: Figure S8**. Maximum likelihood tree of XCP1 and related proteins. The protein sequences of OG0003401 (red) and OG0003952 (green) are obtained from van Velzen et al. (2018). Species include arabidopsis (Athaliana), *Eucalyptus grandis* (Egrandis), *Fragaria vesca* (Fvesca), *Glycine max* (Gmax), medicago (Mtruncatula), *Populus trichocarpa* (Ptrichocarpa), *Parasponia andersonii* (Pan) and *Trema orientalis* (Tor). Numbers at the branches indicate support from 1000 ultrafast bootstrap replicates. OG0003401 containing CEP1 was used as outgroup. (DOCX)

**Additional file 9: Figure S9**. Maximum likelihood tree of APL and related proteins. The protein sequences of OG0009526 (red) and OG0006786 (green) are obtained from van Velzen et al. (2018). Species include arabidopsis (Athaliana), *Eucalyptus grandis* (Egrandis), *Fragaria vesca* (Fvesca), *Glycine max* (Gmax), medicago (Mtruncatula), *Populus trichocarpa* (Ptrichocarpa), *Parasponia andersonii* (Pan) and *Trema orientalis* (Tor). Numbers at the branches indicate support from 1000 ultrafast bootstrap replicates. OG0009526 containing sequences highly homologous to MtAPL was used as outgroup. (DOCX)

**Additional file 10: Figure S10**. The NAC domain transcription factors *MtVND6* and *MtVND7*, but not *MtSND1*, are lower expressed in zone 3 of *Mtnoot1* tnk507 roots when compared to wild-type. The data represent means + SEM of three independent experiments. Student *t*-test was performed to assess significant differences (ns: not significant). (DOCX)

**Additional file 11: Table S1**. qRT-PCR primers used in this study. (DOCX)

## Data Availability

The datasets supporting the conclusions of this research and materials used in this research are available by contacting with the corresponding author (rene.geurts@wur.nl).

## Abbreviations

AZ: Abscission zone
DAG: Days after germination
DMSO: Dimethyl sulfoxide
EDZ: Elongation/differentiation zone
LCO: Lipo-chitooligosaccharide
nt: Nucleotide
PBS: Sodium phosphate buffer
qRT-PCR: Quantitative reverse transcriptase polymerase chain reaction
RAM: Root apical meristem
TZ: Transition zone

## References

[1] Xiao TT, Schilderink S, Moling S, Deinum EE, Kondorosi E, Franssen H (2014). Fate map of *Medicago truncatula* root nodules. Development.

[2] Schauser L, Roussis A, Stiller J, Stougaard J (1999). A plant regulator controlling development of symbiotic root nodules. Nature..

[3] Marsh JF, Rakocevic A, Mitra RM, Brocard L, Sun J, Eschstruth A (2007). *Medicago truncatula* NIN is essential for Rhizobial-independent nodule organogenesis induced by autoactive calcium/Calmodulin-dependent protein kinase. Plant Physiol.

[4] Desbrosses GJ, Stougaard J (2011). Root nodulation: a paradigm for how plant-microbe symbiosis influences host developmental pathways. Cell Host and Microbe.

[5] Yano K, Yoshida S, Müller J, Singh S, Banba M, Vickers K (2008). CYCLOPS, a mediator of symbiotic intracellular accommodation. Proc Natl Acad Sci U S A.

[6] Vernié T, Kim J, Frances L, Ding Y, Sun J, Guan D (2015). The NIN transcription factor coordinates diverse nodulation programs in different tissues of the *Medicago truncatula* root. Plant Cell.

[7] Van Zeijl A (2015). Op Den camp RHM, Deinum EE, Charnikhova T, Franssen H, Op Den camp HJM, et al. rhizobium Lipo-chitooligosaccharide signaling triggers accumulation of Cytokinins in *Medicago truncatula* roots. Mol Plant.

[8] Soyano T, Kouchi H, Hirota A, Hayashi M (2013). NODULE INCEPTION directly targets *NF-Y* subunit genes to regulate essential processes of root nodule development in *Lotus japonicus*. PLoS Genet.

[9] Liu J, Rutten L, Limpens E, van der Molen T, van Velzen R, Chen R (2019). A remote *cis*-regulatory region is required for *NIN* expression in the Pericycle to initiate nodule primordium formation in *Medicago truncatula*. Plant Cell.

[10] Couzigou J-MJ, Zhukov V, Mondy S, Abu el Heba G, Cosson V, THN E (2012). NODULE ROOT and COCHLEATA maintain nodule development and are legume Orthologs of Arabidopsis BLADE-ON-PETIOLE genes. Plant Cell.

[11] Magne K, George J, Berbel Tornero A, Broquet B, Madueño F, Andersen SU (2018). *Lotus japonicus NOOT-BOP-COCH-LIKE1* is essential for nodule, nectary, leaf and flower development. Plant J.

[12] Magne K, Couzigou J-M, Schiessl K, Liu S, George J, Zhukov V (2018). MtNODULE ROOT1 and MtNODULE ROOT2 are essential for indeterminate nodule identity. Plant Physiol.

[13] Benedito VA, Torres-Jerez I, Murray JD, Andriankaja A, Allen S, Kakar K (2008). A gene expression atlas of the model legume *Medicago truncatula*. Plant J.

[14] Holmes P, Goffard N, Weiller GF, Rolfe BG, Imin N (2008). Transcriptional profiling of *Medicago truncatula* meristematic root cells. BMC Plant Biol.

[15] Aida M, Tasaka M (2006). Genetic control of shoot organ boundaries. Curr Opin Plant Biol.

[16] Khan M, Xu H, Hepworth SR (2014). BLADE-ON-PETIOLE genes: setting boundaries in development and defense. Plant Sci.

[17] Žádníková P, Simon R (2014). How boundaries control plant development. Curr Opin Plant Biol.

[18] Hepworth SR, Pautot VA (2015). Beyond the divide: boundaries for patterning and stem cell regulation in plants. Front Plant Sci.

[19] Wang Q, Hasson A, Rossmann S, Theres K (2016). *Divide et impera*: boundaries shape the plant body and initiate new meristems. New Phytol.

[20] Jun JH, Ha CM, Fletcher JC (2010). BLADE-ON-PETIOLE1 coordinates organ determinacy and axial polarity in *Arabidopsis* by directly activating ASYMMETRIC LEAVES2. Plant Cell.

[21] Bell E. M., Lin W.-c., Husbands A. Y., Yu L., Jaganatha V., Jablonska B., Mangeon A., Neff M. M., Girke T., Springer P. S. (2012). Arabidopsis LATERAL ORGAN BOUNDARIES negatively regulates brassinosteroid accumulation to limit growth in organ boundaries. Proceedings of the National Academy of Sciences.

[22] Ha CM, Kim G-T, Kim BC, Jun JH, Soh MS, Ueno Y (2003). The *BLADE-ON-PETIOLE 1* gene controls leaf pattern formation through the modulation of meristematic activity in *Arabidopsis*. Development.

[23] Ha CM, Jun JH, Nam HG, Fletcher JC (2007). *BLADE-ON-PETIOLE1* and *2* control *Arabidopsis* lateral organ fate through regulation of LOB domain and Adaxial-Abaxial polarity genes. Plant Cell.

[24] McKim SM, Stenvik G-E, Butenko MA, Kristiansen W, Cho SK, Hepworth SR (2008). The *BLADE-ON-PETIOLE* genes are essential for abscission zone formation in *Arabidopsis*. Development.

[25] Couzigou JM, Magne K, Mondy S, Cosson V, Clements J, Ratet P (2016). The legume *NOOT-BOP-COCH-LIKE (NBCL)* genes are conserved regulators of abscission, a major agronomical trait in cultivated crops. New Phytol.

[26] Ichihashi Y, Kawade K, Usami T, Horiguchi G, Takahashi T, Tsukaya H (2011). Key proliferative activity in the junction between the leaf blade and leaf Petiole of Arabidopsis. Plant Physiol.

[27] Liebsch D, Sunaryo W, Holmlund M, Norberg M, Zhang J, Hall HC (2014). Class I KNOX transcription factors promote differentiation of cambial derivatives into xylem fibers in the *Arabidopsis* hypocotyl. Development.

[28] Woerlen N, Allam G, Popescu A, Corrigan L, Pautot V, Hepworth SR (2017). Repression of *BLADE-ON-PETIOLE* genes by KNOX homeodomain protein BREVIPEDICELLUS is essential for differentiation of secondary xylem in Arabidopsis root. Planta.

[29] He J, Benedito VA, Wang M, Murray JD, Zhao PX, Tang Y (2009). The *Medicago truncatula* gene expression atlas web server. BMC Bioinformatics.

[30] Dello Ioio R, Linhares FS, Scacchi E, Casamitjana-Martinez E, Heidstra R, Costantino P (2007). Cytokinins determine *Arabidopsis* root-meristem size by controlling cell differentiation. Curr Biol.

[31] Ioio RD, Nakamura K, Moubayidin L, Perilli S, Taniguchi M, Morita MT (2008). A genetic framework for the control of cell division and differentiation in the root meristem. Science.

[32] Khan M, Xu M, Murmu J, Tabb P, Liu Y, Storey K (2012). Antagonistic interaction of BLADE-ON-PETIOLE1 and 2 with BREVIPEDICELLUS and PENNYWISE regulates Arabidopsis inflorescence architecture. Plant Physiol.

[33] Schuetz M, Smith R, Ellis B (2013). Xylem tissue specification, patterning, and differentiation mechanisms. J Exp Bot.

[34] Lars Hennig, Köhler C. Plant Developmental Biology Methods and Protocols 2010.

[35] Zhong R, Lee C, Zhou J, McCarthy RL, Ye Z-H (2008). A battery of transcription factors involved in the regulation of secondary Cell Wall biosynthesis in *Arabidopsis*. Plant Cell Online.

[36] Ohashi-Ito K, Oda Y, Fukuda H (2010). *Arabidopsis* VASCULAR-RELATED NAC-DOMAIN6 directly regulates the genes that govern programmed cell death and secondary wall formation during xylem differentiation. Plant Cell.

[37] Zhong R, Lee C, Ye Z-H (2010). Global analysis of direct targets of secondary wall NAC master switches in *Arabidopsis*. Mol Plant.

[38] Bonke M, Thitamadee S, Mähönen AP, Hauser MT, Helariutta Y (2003). APL regulates vascular tissue identity in *Arabidopsis*. Nature.

[39] Rost TL (2011). The organization of roots of dicotyledonous plants and the positions of control points. Ann Bot.

[40] Kong X, Liu G, Liu J, Ding Z (2018). The root transition zone: a hot spot for signal crosstalk. Trends Plant Sci.

[41] Campbell L, Turner S (2017). Regulation of vascular cell division. J Exp Bot.

[42] Ruonala R, Ko D, Helariutta Y (2017). Genetic networks in plant vascular development. Annu Rev Genet.

[43] Matsumoto-Kitano M, Kusumoto T, Tarkowski P, Kinoshita-Tsujimura K, Vaclavikova K, Miyawaki K (2008). Cytokinins are central regulators of cambial activity. Proc Natl Acad Sci.

[44] Smetana O, Mäkilä R, Lyu M, Amiryousefi A, Sánchez Rodríguez F, Wu M-F (2019). High levels of auxin signalling define the stem-cell organizer of the vascular cambium. Nature.

[45] Boivin S, Fonouni-Farde C, Frugier F (2016). How Auxin and Cytokinin Phytohormones Modulate Root Microbe Interactions. Front Plant Sci.

[46] Gamas P, Brault M, Jardinaud MF, Frugier F (2017). Cytokinins in symbiotic nodulation: when, where, what for?. Trends Plant Sci.

[47] Kohlen W, Ng JLP, Deinum EE, Mathesius U (2018). Auxin transport, metabolism, and signalling during nodule initiation: indeterminate and determinate nodules. J Exp Bot.

[48] Gonzalez-Rizzo S, Crespi M, Frugier F (2006). The *Medicago truncatula* CRE1 cytokinin receptor regulates lateral root development and early symbiotic interaction with *Sinorhizobium meliloti*. Plant Cell.

[49] Nadzieja M, Kelly S, Stougaard J, Reid D (2018). Epidermal auxin biosynthesis facilitates rhizobial infection in *Lotus japonicus*. Plant J.

[50] Hoffmann B, Trinh TH, Leung J, Kondorosi A, Kondorosi E (1997). A new *Medicago truncatula* line with superior in vitro regeneration, transformation, and symbiotic properties isolated through cell culture selection. Mol Plant-Microbe Interact.

[51] Limpens E, Ramos J, Franken C, Raz V, Compaan B, Franssen H (2004). RNA interference in *Agrobacterium rhizogenes*-transformed roots of *Arabidopsis* and *Medicago truncatula*. J Exp Bot.

[52] Fahraeus G (1957). The infection of clover root hairs by nodule bacteria studied by a simple glass slide technique. J Gen Microbiol.

[53] Kulikova O, Franken C, Bisseling T (2018). Methods in Molecular Biology. In situ hybridization method for localization of mRNA molecules in medicago tissue sections.

[54] Katsushima K, Natsume A, Ohka F, Shinjo K, Hatanaka A, Ichimura N (2016). Targeting the Notch-regulated non-coding RNA TUG1 for glioma treatment. Nat Commun.

[55] Osteen JD, Herzig V, Gilchrist J, Emrick JJ, Zhang C, Wang X (2016). Selective spider toxins reveal a role for the Nav1.1 channel in mechanical pain. Nature.

[56] Roux B, Rodde N, Jardinaud MF, Timmers T, Sauviac L, Cottret L (2014). An integrated analysis of plant and bacterial gene expression in symbiotic root nodules using laser-capture microdissection coupled to RNA sequencing. Plant J.

[57] van Velzen R, Holmer R, Bu F, Rutten L, van Zeijl A, Liu W (2018). Comparative genomics of the nonlegume *Parasponia* reveals insights into evolution of nitrogen-fixing rhizobium symbioses. Proc Natl Acad Sci U S A.

[58] Katoh K (2002). MAFFT: a novel method for rapid multiple sequence alignment based on fast Fourier transform. Nucleic Acids Res.

[59] Trifinopoulos J, Nguyen LT, von Haeseler A, Minh BQ (2016). W-IQ-TREE: a fast online phylogenetic tool for maximum likelihood analysis. Nucleic Acids Res.

[60] Kalyaanamoorthy S, Minh BQ, Wong TKF, Von Haeseler A, Jermiin LS (2017). ModelFinder: fast model selection for accurate phylogenetic estimates. Nat Methods.

[61] Minh BQ, Nguyen MAT, Von Haeseler A (2013). Ultrafast approximation for phylogenetic bootstrap. Mol Biol Evol.

