# Supplementary figures and images for: The Medicago truncatula nodule identity gene MtNOOT1 is required for coordinated apical-basal development of the root
Source: BMC Plant Biol. 2019 Dec 19;19:571. doi: 10.1186/s12870-019-2194-z (PMC6923920; doi:10.1186/s12870-019-2194-z)

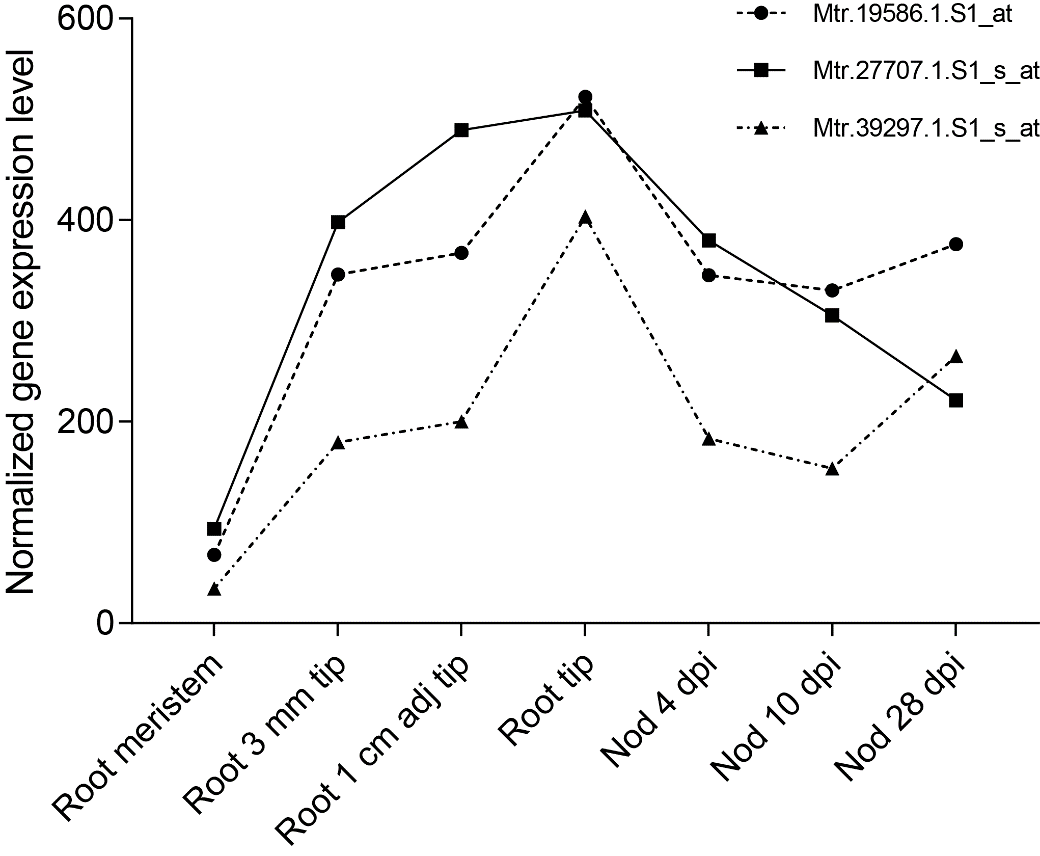

Supplement: Supplementary file 1 — Additional file 1: Figure S1. Medicago MtNOOT1 is expressed in the root tip. Expression profiles are derived from the Medicago truncatula Gene Expression Atlas [29]. MtNOOT1 is targeted by the probe-sets Mtr.19586.1.S1_at, Mtr.27707.1.S1_s_at, and Mtr.39297.1.S1_s_at. Root 3 mm tip: 3 mm root tip [14]; adj tip: 1 cm root segment adjacent to 3 mm root tip [14]; Nod: nodules, all nodule samples are derived from [13]; dpi: days post inoculation. (DOCX) [file 12870_2019_2194_MOESM1_ESM.docx]

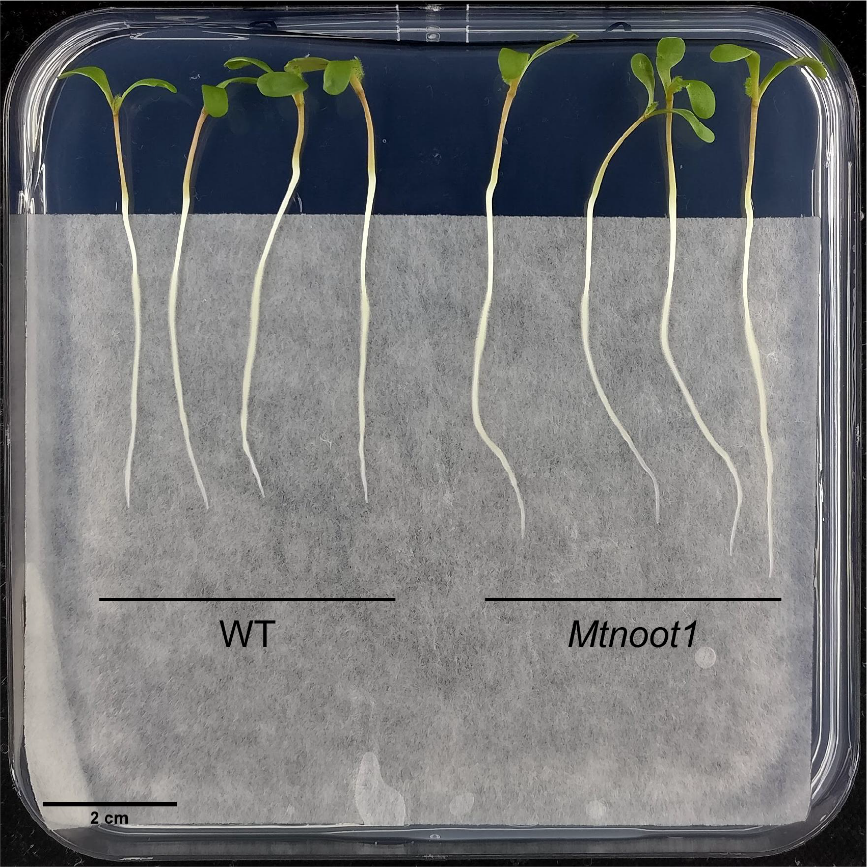

Supplement: Supplementary file 2 — Additional file 2: Figure S2. The primary root Mtnoot1 tnk507 mutant is longer than wild-type. Representative seedlings at 6 DAG are presented. (DOCX) [file 12870_2019_2194_MOESM2_ESM.docx]

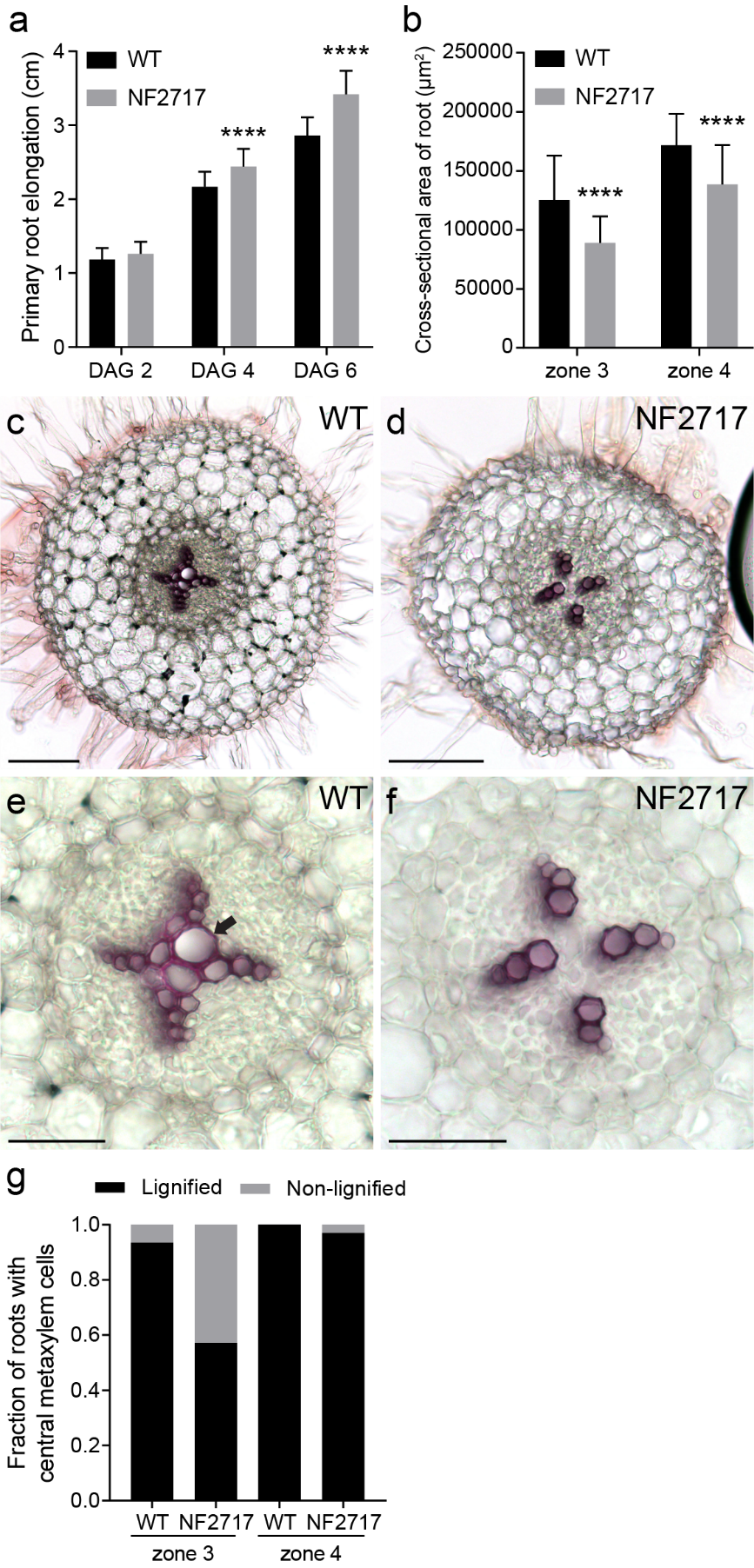

Supplement: Supplementary file 3 — Additional file 3: Figure S3. The Mtnoot1 NF2717 mutant allele shows a similar phenotype as the Mtnoot1 tnk507 allele. a Root length of the medicago Mtnoot1 mutant (NF2717) is markedly longer at 4 DAG and 6 DAG when compared to primary roots of wild-type medicago seedlings (WT). b The cross-sectional area is significantly reduced in the medicago Mtnoot1 mutant (NF2717) at zone 3 and zone 4. The data represent means + SD of two independent experiments, each experiment contains 15–20 roots. Student t-test was performed to assess significant differences (****: P < 0.0001). Representative root cross sections of wild-type (c, e) and Mtnoot1 (NF2717) (d, f) vascular bundle at zone 3 stained with phloroglucinol-HCl to demonstrate lignin deposition at 6 DAG. Black arrow marks lignified metaxylem cells, which are not found in the Mtnoot1 mutant (NF2717). g Vascular xylem differentiation is delayed in the Mtnoot1 (NF2717) primary root at 6 DAG. The fraction of roots with lignified central metaxylem cells in zone 3 and zone 4 is decreased in the Mtnoot1 mutant when compared with wild-type seedlings. The presented data combines two independent experiments, each experiment contains 15–18 roots. Scale bar: 50 μm (c, d), 100 μm (e, f). (DOCX) [file 12870_2019_2194_MOESM3_ESM.docx]

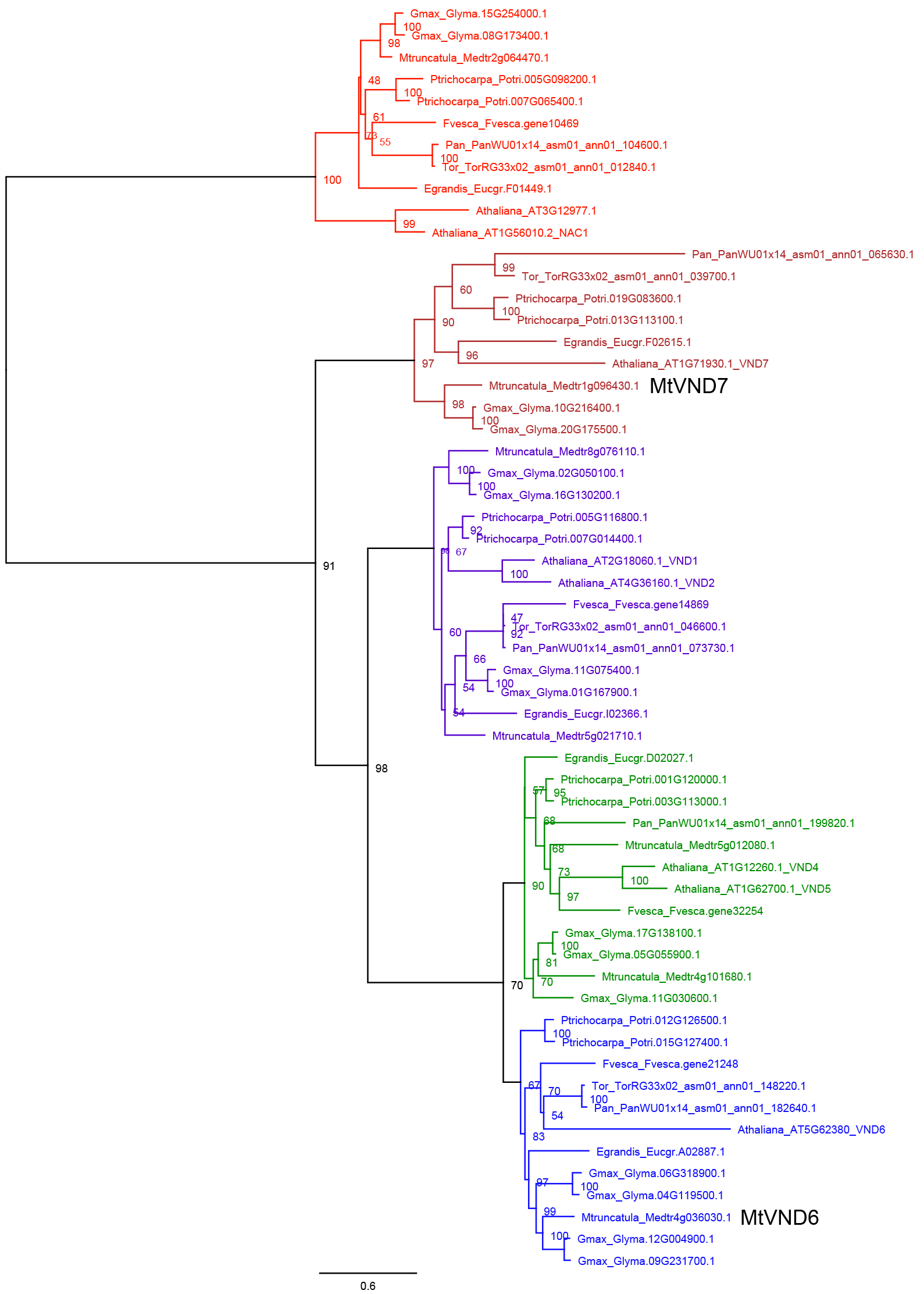

Supplement: Supplementary file 4 — Additional file 4: Figure S4. Maximum likelihood tree of VND6, VND7 and related proteins. The protein sequences of OG0006787 (red), OG0009959 (dark red), OG0004118 (purple), OG0001465 (green and blue) are obtained from van Velzen et al. (2018), except VND6, which was not included in OG0001465. Species include arabidopsis (Athaliana), Eucalyptus grandis (Egrandis), Fragaria vesca (Fvesca), Glycine max (Gmax), medicago (Mtruncatula), Populus trichocarpa (Ptrichocarpa), Parasponia andersonii (Pan) and Trema orientalis (Tor). Numbers at to the branches indicate support from 1000 ultrafast bootstrap replicates. OG0006787 including NAC1 was used as outgroup. (DOCX) [file 12870_2019_2194_MOESM4_ESM.docx]

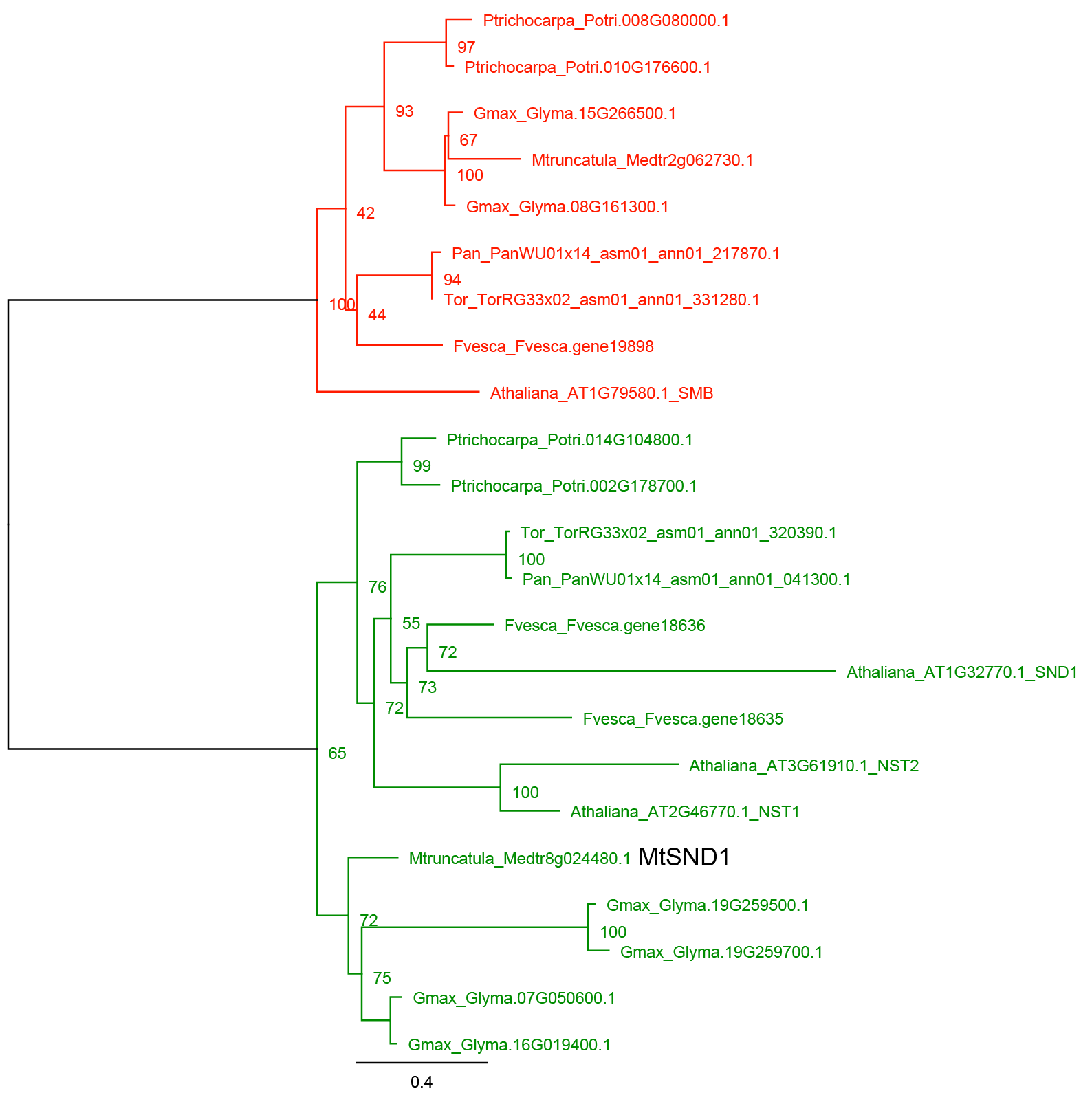

Supplement: Supplementary file 5 — Additional file 5: Figure S5. Maximum likelihood tree of SND1 and related proteins. The protein sequences of OG0009898 (red) and OG0001875 (green) are obtained from van Velzen et al. (2018). Species include arabidopsis (Athaliana), Eucalyptus grandis (Egrandis), Fragaria vesca (Fvesca), Glycine max (Gmax), medicago (Mtruncatula), Populus trichocarpa (Ptrichocarpa), Parasponia andersonii (Pan) and Trema orientalis (Tor). Numbers at to the branches indicate support from 1000 ultrafast bootstrap replicates. OG0009898 containing SMB was used as outgroup. (DOCX) [file 12870_2019_2194_MOESM5_ESM.docx]

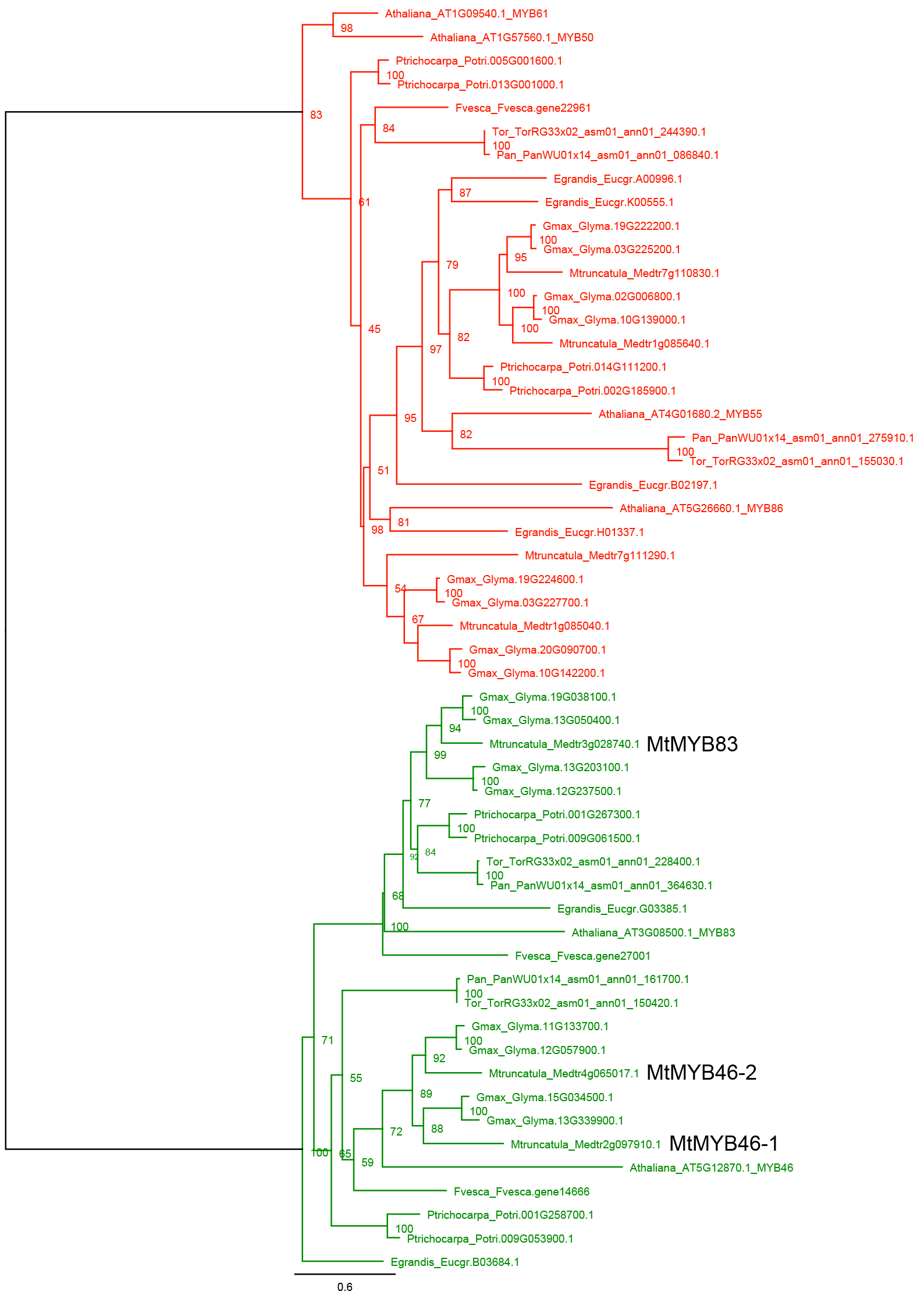

Supplement: Supplementary file 6 — Additional file 6: Figure S6. Maximum likelihood tree of MYB46, MYB83 and related proteins. The protein sequences of OG0000857 (red) and OG0001270 (green) are obtained from van Velzen et al. (2018). Species include arabidopsis (Athaliana), Eucalyptus grandis (Egrandis), Fragaria vesca (Fvesca), Glycine max (Gmax), medicago (Mtruncatula), Populus trichocarpa (Ptrichocarpa), Parasponia andersonii (Pan) and Trema orientalis (Tor). Numbers at the branches indicate support from 1000 ultrafast bootstrap replicates. OG0000857 containing MYB50 was used as outgroup. (DOCX) [file 12870_2019_2194_MOESM6_ESM.docx]

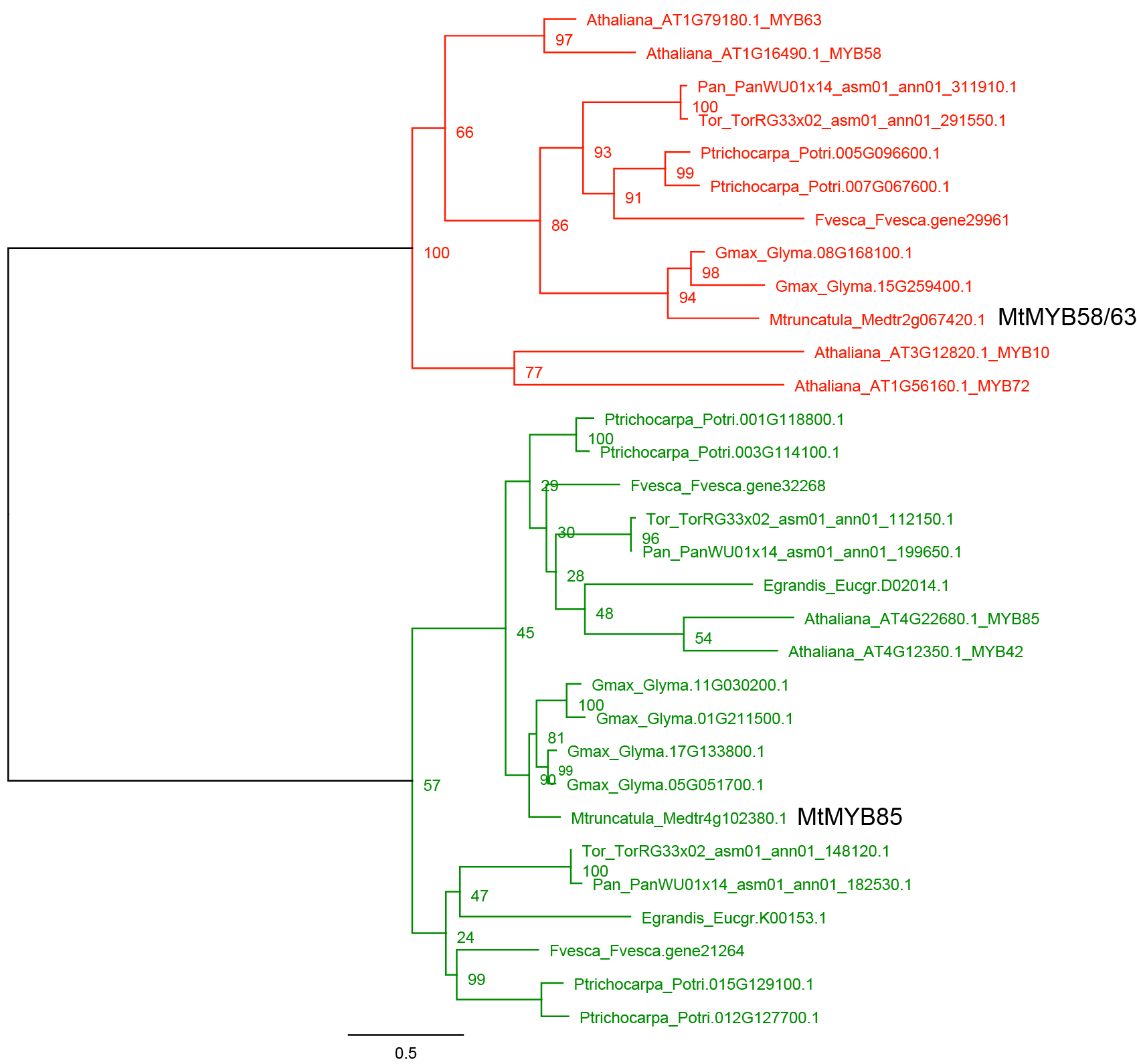

Supplement: Supplementary file 7 — Additional file 7: Figure S7. Maximum likelihood tree of MYB58, MYB63 and MYB85 proteins. The protein sequences of OG0005384 (red) and OG0002420 (green) are obtained from van Velzen et al. (2018). Species include arabidopsis (Athaliana), Eucalyptus grandis (Egrandis), Fragaria vesca (Fvesca), Glycine max (Gmax), medicago (Mtruncatula), Populus trichocarpa (Ptrichocarpa), Parasponia andersonii (Pan) and Trema orientalis (Tor). Numbers at the branches indicate support from 1000 ultrafast bootstrap replicates. OG0005384 containing MYB58 and MYB63 was used as outgroup. (DOCX) [file 12870_2019_2194_MOESM7_ESM.docx]

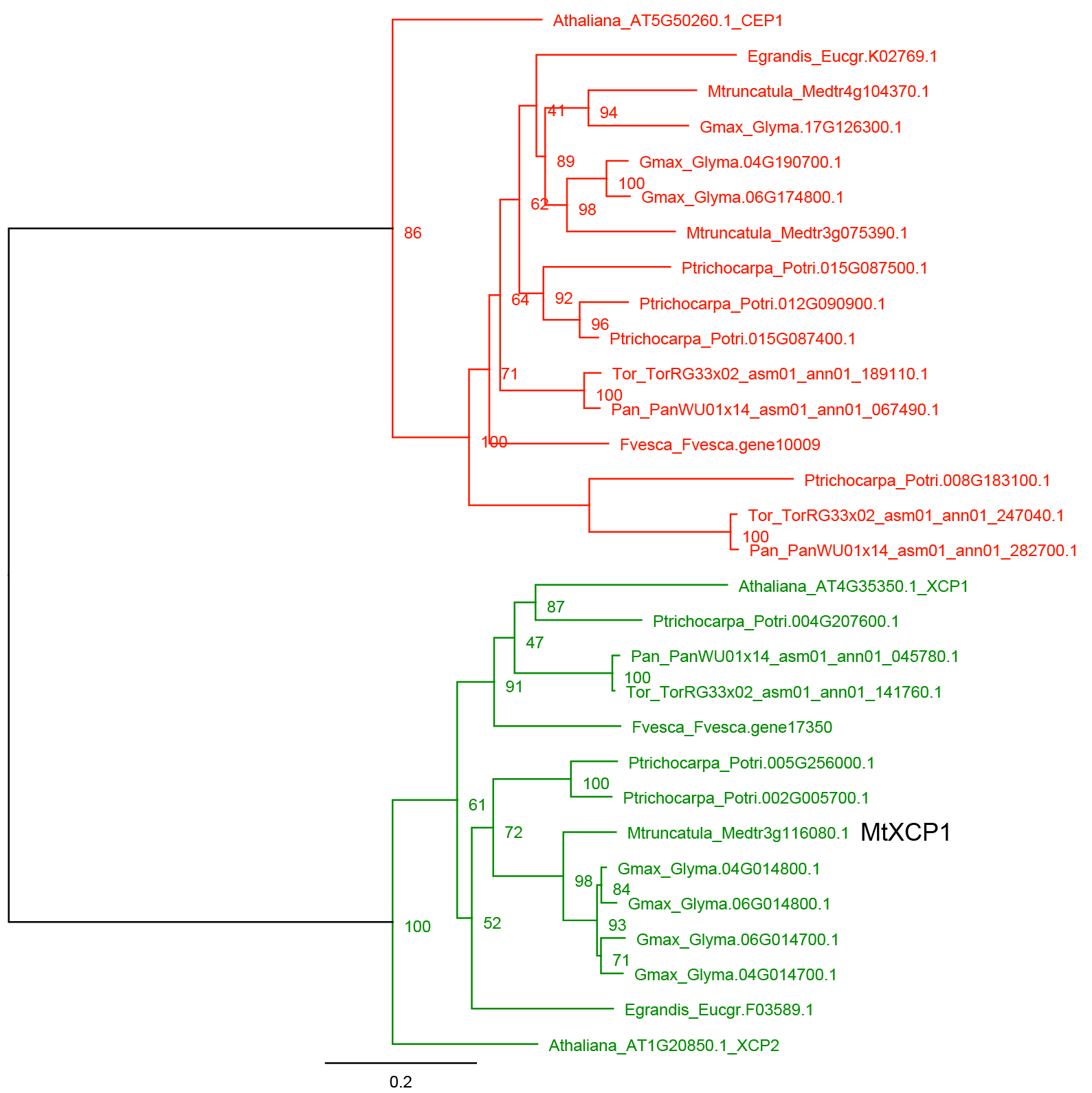

Supplement: Supplementary file 8 — Additional file 8: Figure S8. Maximum likelihood tree of XCP1 and related proteins. The protein sequences of OG0003401 (red) and OG0003952 (green) are obtained from van Velzen et al. (2018). Species include arabidopsis (Athaliana), Eucalyptus grandis (Egrandis), Fragaria vesca (Fvesca), Glycine max (Gmax), medicago (Mtruncatula), Populus trichocarpa (Ptrichocarpa), Parasponia andersonii (Pan) and Trema orientalis (Tor). Numbers at the branches indicate support from 1000 ultrafast bootstrap replicates. OG0003401 containing CEP1 was used as outgroup. (DOCX) [file 12870_2019_2194_MOESM8_ESM.docx]

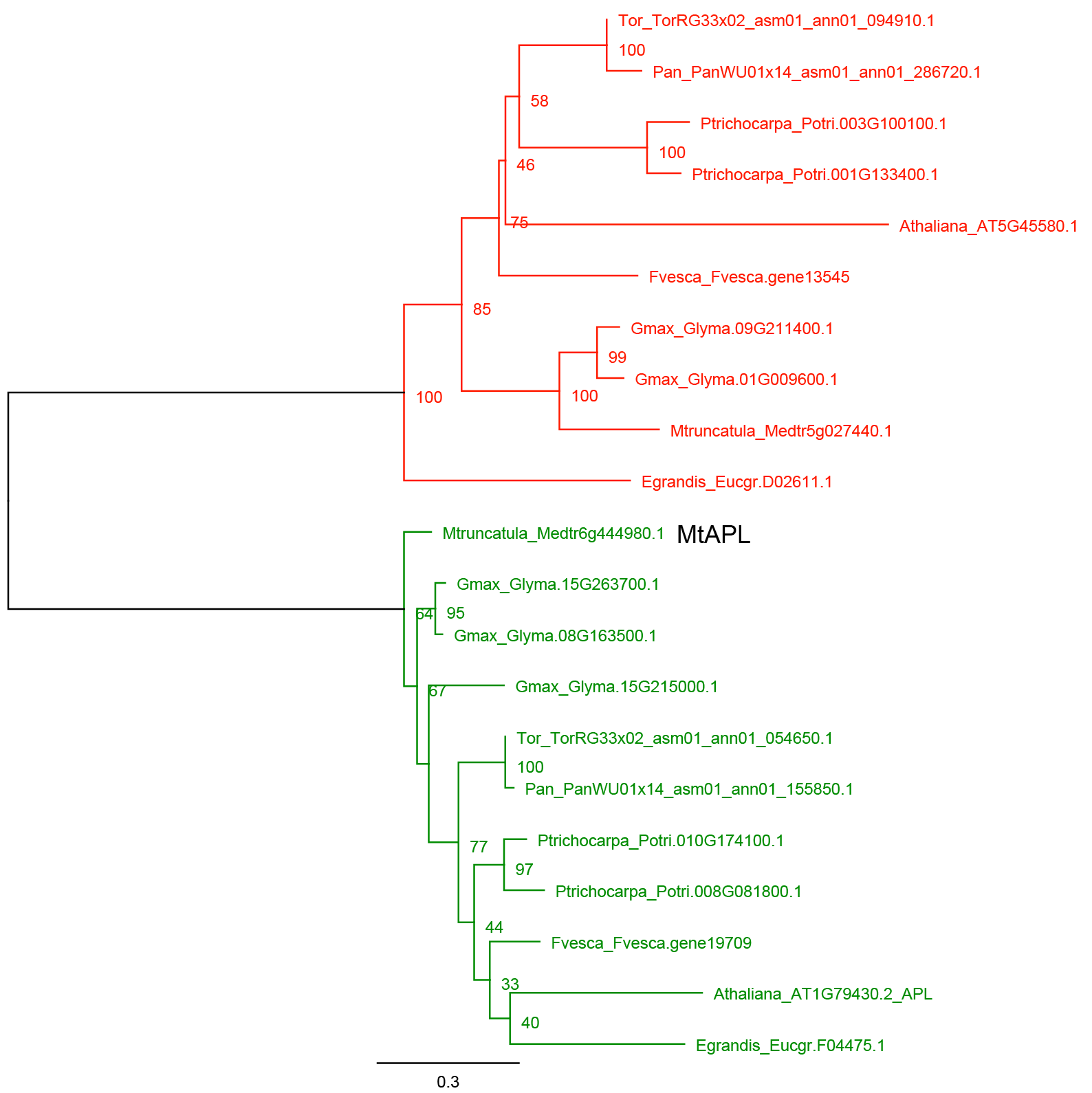

Supplement: Supplementary file 9 — Additional file 9: Figure S9. Maximum likelihood tree of APL and related proteins. The protein sequences of OG0009526 (red) and OG0006786 (green) are obtained from van Velzen et al. (2018). Species include arabidopsis (Athaliana), Eucalyptus grandis (Egrandis), Fragaria vesca (Fvesca), Glycine max (Gmax), medicago (Mtruncatula), Populus trichocarpa (Ptrichocarpa), Parasponia andersonii (Pan) and Trema orientalis (Tor). Numbers at the branches indicate support from 1000 ultrafast bootstrap replicates. OG0009526 containing sequences highly homologous to MtAPL was used as outgroup. (DOCX) [file 12870_2019_2194_MOESM9_ESM.docx]

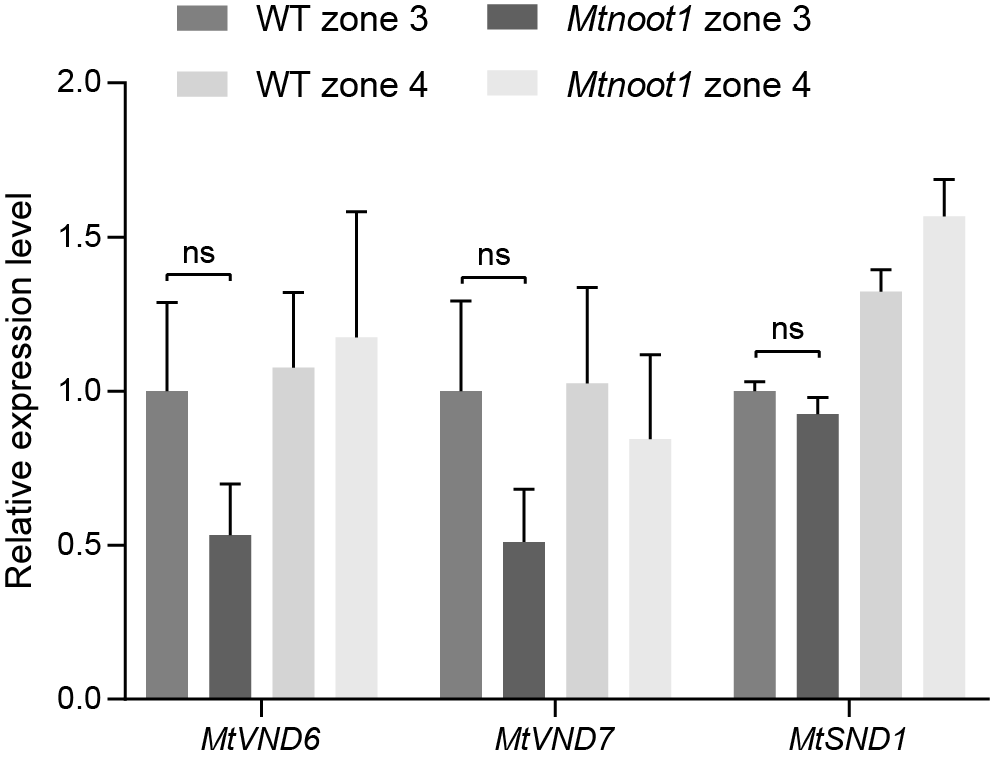

Supplement: Supplementary file 10 — Additional file 10: Figure S10. The NAC domain transcription factors MtVND6 and MtVND7, but not MtSND1, are lower expressed in zone 3 of Mtnoot1 tnk507 roots when compared to wild-type. The data represent means + SEM of three independent experiments. Student t-test was performed to assess significant differences (ns: not significant). (DOCX) [file 12870_2019_2194_MOESM10_ESM.docx]
