# Supplementary material for: The Medicago truncatula nodule identity gene MtNOOT1 is required for coordinated apical-basal development of the root
Source: BMC Plant Biol. 2019 Dec 19;19:571. doi: 10.1186/s12870-019-2194-z (PMC6923920; doi:10.1186/s12870-019-2194-z)
Supplement: Supplementary file 11 — Additional file 11: Table S1. qRT-PCR primers used in this study. (DOCX) [file 12870_2019_2194_MOESM11_ESM.docx]

| **Name** | **Gene ID** | **Sequence** |
| --- | --- | --- |
| MtACT2-F | Medtr2g008050 | CAGATGTGGATCTCCAAGGGTGA |
| MtACT2-R |  | TGACTGAAATATGGCACAAGACTGAGA |
| MtAPL-F | Medtr6g444980 | ACTTGAAAGACCTTCACCAAGA |
| MtAPL-R |  | GAACCTCTACCTTGTGCCATAG |
| MtMYB46-1-F | Medtr2g097910 | ACTTCATCATCAACATCTCCATCA |
| MtMYB46-1-R |  | ACTATGATCCATCACAGGCAAC |
| MtMYB46-2-F | Medtr4g065017 | ACAACTGCAATGTTGTTAGTAAGAG |
| MtMYB46-2-R |  | CTTCCAAATTCCAATCTCCAACAG |
| MtMYB58/63-F | Medtr2g067420 | TCAATCAAATGTTGGTGAAGAGAC |
| MtMYB58/63-R |  | CACCTACTACTCCAAACTCATTCT |
| MtMYB83-F | Medtr3g028740 | AAAGCCATAACAACCACTTCAA |
| MtMYB83-R |  | TCTCCTTGCCCATGATTTCC |
| MtMYB85-F | Medtr4g102380 | GATAGCATTTGCAGTGACGATTC |
| MtMYB85-R |  | CTGCTATAGGTGTAGTGTCCATTT |
| MtNOOT1-F | Medtr7g090020 | GTTTAGTCCACGCACACAGA |
| MtNOOT1-R |  | CAGAAACTGAAGCATCAACAAGAA |
| MtSND1-F | Medtr8g024480 | CAACACACGCCAGAACTACTA |
| MtSND1-R |  | AGAAGACGACAACAAGGATGAA |
| MtVND6-F | Medtr4g036030 | GTTGCTTCACAACTTAGTCAAGAT |
| MtVND6-R |  | GGCACCATTTCTTGTTTCTCC |
| MtVND7-F | Medtr1g096430 | AGCATGCCATTGATGATACCT |
| MtVND7-R |  | TGAATCAGGAAAGCAACCTAAGA |
| MtXCP1-F | Medtr3g116080 | CAGGCAGAGATTTCCAGTTCTA |
| MtXCP1-R |  | CCAAACCCTTTGATGTACCATATC |
